# Heterosis unveiled in root-related traits and saikosaponins content between triploid F_1_ hybrids and parental *Bupleurum chinense* DC.

**DOI:** 10.3389/fpls.2026.1736464

**Published:** 2026-02-12

**Authors:** Chuanxin Mo, Wenshuai Chen, Zhuolin Lv, Jun Zhao, Yuchan Li, Qiannan Shi, Kaimi Dong, Zhen Wei, Zeru Yu, Xueling Wang, Chao Xin, Zhen Ni, Ma Yu, Hua Chen

**Affiliations:** 1 College of Life Sciences and Agri-forestry, Southwest University of Science and Technology, Mianyang, Sichuan, China; 2 Institute of Plateau Biology of Xizang Autonomous Region, Lhasa, Xizang, China; 3 Jining Polytechnic, Jining, Shandong, China

**Keywords:** *Bupleurum chinense*, heterosis, karyotype, polyploid breeding, saikosaponins

## Abstract

The root biomass and saikosaponins yield of *Bupleurum chinense* DC. are crucial factors determining its economic value. This study developed triploid F_1_ hybrid materials of the *B. chinense* by crossing a diploid (2n=2x_1_ = 12, x_1_ = 6) maternal parent with a tetraploid (2n=4x_2_ = 20, x_2_ = 5) paternal parent. The resulting hybrids exhibited a stable intergenomic karyotype (2n=x_1_+2x_2_ = 16) and significant heterosis. Two-year field trials confirmed strong over-dominance in root architecture, with root dry weight exceeding that of the diploid and tetraploid parents by 37.3% and 166.5%, respectively. The yield of the bioactive compounds saikosaponin A (13.42 mg) and D (13.20 mg) increased by an average of 60.7% and 57.5%, respectively, compared to the diploid parent, highlighting substantial potential for pharmaceutical development. Based on the transcriptome comparison in the seedling and maturity stage of the root, the remarkable heterosis might be supported by unique genomic architecture and sophisticated transcriptional reprogramming. The intergenomic imbalance, likely provide a stable foundation for heterosis by facilitating functional compartmentalization and synergistic interaction between the parental subgenomes. Transcriptome analysis revealed that the heterotic traits were arranged by a complex relationship of gene networks: root morphology was optimized through the additive and transgressive expression of hormone signaling genes, while the enhanced synthesis of saikosaponins was driven by the synergistic expression of key biosynthetic genes. This research provides a novel strategy for exceed conventional plant breeding by demonstrating engineered genomic asymmetry, specifically intergenomic triploid and could help to unlock superior and stable heterosis.

## 1 Introduction

Radix Bupleuri, the dry root of plants in the *Bupleurum* genus, has been extensively employed in traditional Chinese medicine for more than 2000 years (Ashour and Wink, 2011; Teng et al., 2023; Zeng et al., 2023). Species in this genus are widely distributed across East and Central Asia, North America, North Africa, and Europe (Pan, 2006; Zeng et al., 2023). According to the Chinese Pharmacopoeia (2025), the official sources of Radix Bupleuri are the dried roots of *Bupleurum chinense* and *B. scorzonerifolium*, which are recognized for their therapeutic effects, including antipyretic, analgesic, hepatoprotective, and antidepressant properties (Teng et al., 2023).

Due to increasing market demand, natural populations of *B. chinense* and *B. scorzonerifolium* have experienced significant depletion in recent years. Therefore, cultivation and breeding efforts have become essential to ensure sustainable supply. Current breeding strategies for *B. chinense* include the introduction of cultivated varieties from other regions, domestication of local wild germplasm, and selection of superior individuals to develop locally adapted varieties (Zheng et al., 2010; Xu et al., 2018). Hybridization breeding is widely employed in agriculture because of its ability to generate offspring exhibiting heterosis (hybrid vigor), where hybrids outperform their parents in yield and growth (Schnable and Springer, 2013; Kim and Zhang, 2018). For instance, heterosis has contributed to yield increases of 3.5–15% in wheat (Whitford et al., 2013), 55% in rice (Yuan, 1994), and up to 200% in canola (Shehzad et al., 2015). Similar effects have been observed in medicinal plants, including *Panax ginseng* (Seo et al., 2019), licorice (*Glycyrrhiza* spp.) (Lee et al., 2017), *Chrysanthemum* (Deng et al., 2012), and Chinese bellflower (*Platycodon grandiflorus*) (Wei et al., 2011). In the genus of *Bupleurum* L., an interspecific F_1_ hybrid between *B. chinense* and *B. marginatum* exhibited significant heterosis in root yield and growth adaptability (Xu, 2019). Interspecific hybrid materials are generally not adopted in commercial medicinal‐material production because their safety, efficacy, and quality stability require rigorous risk assessment. Despite the demonstrated potential of hybridization breeding, polyploid‐based hybrid breeding, particularly triploid formation through asymmetric ploidy crosses, has not yet been systematically explored in *B. chinense*, leaving its effects on growth performance and secondary metabolism largely unknown.

Hybridization leads to the combination of divergent alleles, often resulting in structural rearrangements and epigenetic reprogramming. These molecular events induce heritable changes in gene expression and phenotype, contributing to species diversification and evolution (Jackson, 2017; Nieto Feliner et al., 2020). A commonly observed phenomenon in hybrids is “transcriptome shock,” wherein the merging of distinct parental genomes triggers non-additive gene expression patterns, with F_1_ expression levels deviating from the mid-parent value (Rapp et al., 2009). Among non-additively expressed genes, transgressive expression refers to expression levels that exceed the parental range, while expression-level dominance describes gene expression in the F_1_ that matches one parent but diverges significantly from the other (Wu et al., 2016; Díaz-Valenzuela et al., 2023). Transcriptome shock also entails extensive rewiring of regulatory networks and differential partitioning of parental homeolog expression (Quan et al., 2022).

Saikosaponins, the principal bioactive constituents of *B. chinense*, are pentacyclic triterpenoid saponins synthesized through a multistep biosynthetic pathway (Yu et al., 2020; Sui et al., 2011). The biosynthesis begins with the formation of the triterpenoid backbone, primarily via the cytosolic mevalonate (MVA) pathway, although the plastidial methylerythritol phosphate (MEP) pathway also contributes. The MVA pathway starts from acetyl-CoA and proceeds through a series of enzymatic reactions to generate isopentenyl diphosphate (IPP), a key precursor. IPP is subsequently converted to squalene, which is epoxidized to form 2,3-oxidosqualene. This intermediate is cyclized by oxidosqualene cyclases (OSCs) to yield various triterpenoid skeletons (Xue et al., 2012; Wang et al., 2022). These scaffolds undergo further structural diversification through modifications catalyzed by cytochrome P450 monooxygenases (P450s) and UDP-glycosyltransferases (UGTs), which introduce functional groups and glycosyl residues, respectively, thereby enhancing the bioactivity and chemical diversity of saikosaponins (Zhang et al., 2019; Li et al., 2023). Although heterologous biosynthesis offers a promising platform for the production of complex natural products, it requires comprehensive elucidation of metabolic intermediates and pathway enzymes (Park et al., 2020). The objectives of the present study were (a) to compare root morphological traits and saikosaponin profiles between F_1_ hybrids and their parents; (b) to investigate the genetic and transcriptomic differences using RNA sequencing; and (c) to identify heterosis- regulating root development and saikosaponin biosynthesis.

## 2 Materials and methods

### 2.1 Plant materials and hybridization

**Figure 1:**
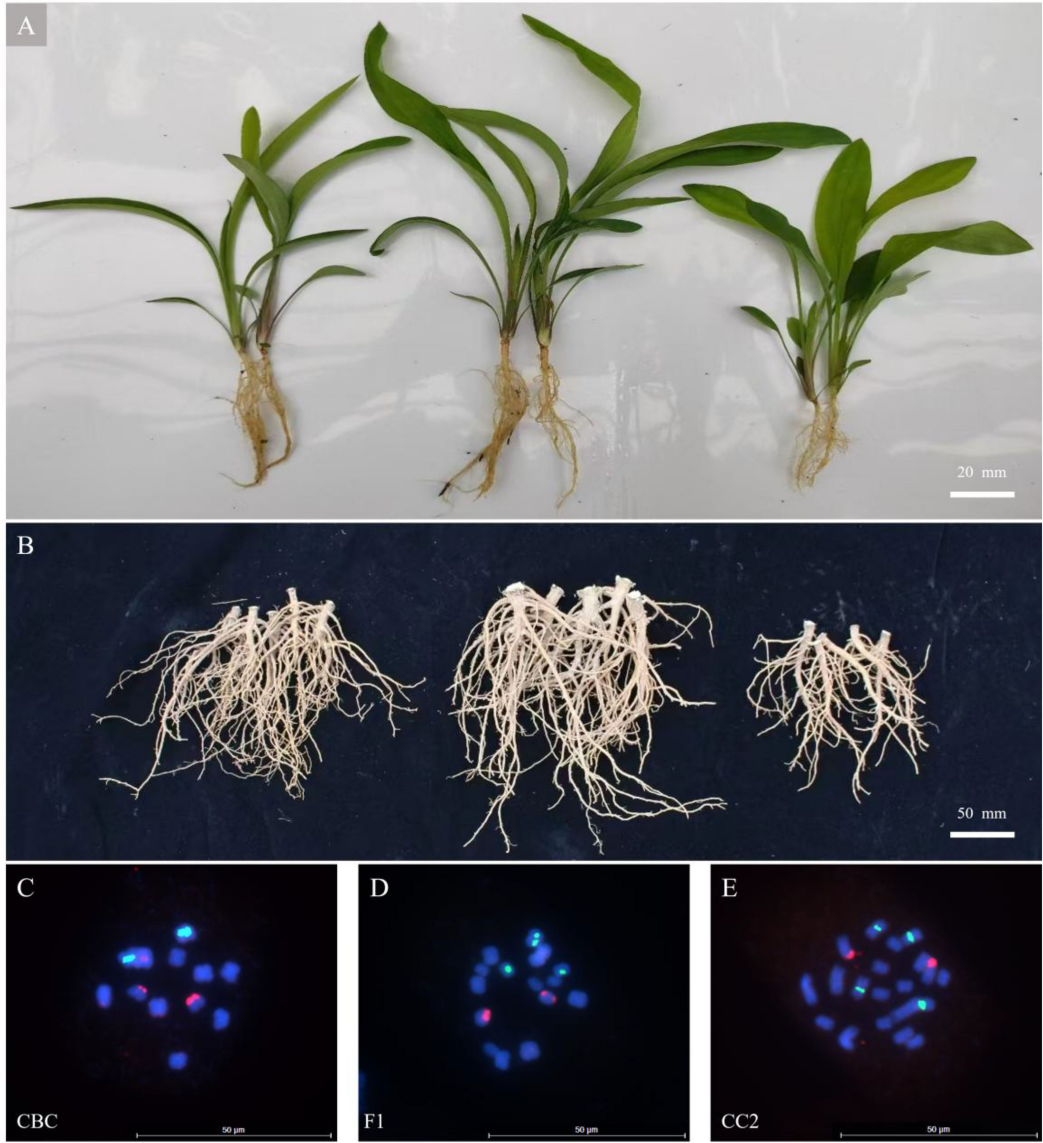
Comparative analysis of root biomass and karyotypes in F_1_ hybrids and parental lines. **(A)** Root architecture phenotypes at seedling stage, from left to right are CBC,F1, and CC2; **(B)** Root architecture phenotypes at maturity stage, from left to right are CBC,F1, and CC2; **(C–E)** Fluorescence *in situ* hybridization patterns of CBC **(C)**, F_1_ hybrid **(D)**, and CC2 **(E)** using 5S rDNA probes (cyan pseudo-color) and 18S rDNA probes (red pseudo-color).

This study utilized an F_1_ generation derived from a cross between two commercial varieties of *B. chinense* (Chuanbeichai #1 and Chuanchai #2), along with both parents (**Figure 1**). Chuanbeichai #1 (hereafter CBC) is a diploid (2n = 2x_1_) cultivar cultivated primarily in northern China (Hebei, Shanxi, and Gansu provinces), is characterized by a high dry root weight and elevated saikosaponin content, but exhibits moderate tolerance to soil waterlogging. The male parent of Chuanchai #2 (hereafter: CC2) is an atuotetraploid (2n = 4x_2_) line introduced from Rongxian county in Sichuan province and was officially registered in 2023. This cultivar shows strong adaptation to the rainy weather of summer in Sichuan province but produces a relatively low dry root yield. Because pistil maturation in CBC lagged behind stamen development, uniformly developed inflorescences were selected and pruned to prevent self-pollination. After the stamens are removed manually, CC2 at full flowering was used as the pollen donor, and F_1_ hybrids were obtained by controlled pollination. Seeds for F_1_ and both cultivars were kindly provided by Dr. Yu from the College of Life Sciences and Agri-forestry, Southwest University of Science and Technology.

### 2.2 Fluorescence *in situ* hybridization

Roots of *B. chinense* (1.5–2.0 mm in length, containing active root apical meristems) were harvested from 7-day-old *in vitro* cultured seedlings. To enrich for metaphase cells, the cultures were exposed to nitrous oxide gas at 1 MPa for two hours to synchronize mitosis. Following treatment, approximately 5 mm root tips were excised, minced, and subjected to enzymatic digestion in a solution of 1% pectolyase and 2% cellulase Onozuka at 37°C for one hour. The resulting cell suspension was centrifuged, and the pellet was resuspended in 90% acetic acid. The suspension was placed onto clean glass slides within a humidified chamber to prevent drying.

Chromosomes were stained with 4′,6-diamidino-2-phenylindole (DAPI), and visualized under a fluorescence microscope (Zeiss LSM880, Germany). For fluorescence *in situ* hybridization (FISH), probes specific to 5S and 18S rDNA repeat sequences were used to detect multiple chromosomal loci. The probes were denatured in hybridization buffer (50% formamide, 10% dextran sulfate, 2×SSC) at 80°C for 10 minutes, hybridized overnight at 42°C, and stringently washed with 2×SSC/0.1% Tween-20 at 55°C. Methodological details are available in Zhang et al. (2022). Fluorescent signals were visualized using confocal microscopy (Leica Microsystems, Wetzlar, Germany). Karyotype construction was based on measurements of relative chromosome length and centromeric index, with a minimum of 15 well-spread metaphase cells analyzed per sample.

### 2.3 Field experiment and phenotypic evaluation

Parental lines and F_1_ hybrids were cultivated at the Longshan Research Farm, Southwest University of Science and Technology, Mianyang, China (31°32′N, 104°42′E) across two growing seasons—sown on March 12^th^, 2021, and March 28^th^, 2022, respectively. The experiment employed a randomized complete block design (RCBD) with three replicates. Each plot (1.5 × 8.0 m) contained two rows with 25 cm row spacing and 3–5 cm plant spacing. Standard agronomic practices were maintained throughout the growing season to ensure uniform growth (Luo, 2024). Plant height was measured in October each year. For each plot, fresh root weight, root length, and taproot diameter were determined from 10 randomly selected plants after harvesting. Roots were oven-dried (Thermo Fisher Scientific, USA) at 120°C for 30 minutes followed by 60°C for 72 hours to determine dry root weight.

On July 18, 2024, parents and F_1_ materials were grown in a controlled-environment greenhouse at Southwest University of Science and Technology using a completely randomized design with three replicates. Greenhouse conditions were maintained at 24°C and 40–60% relative humidity. Plants were individually planted in round pot (10 cm diameter × 11 cm heigh), with 10 plants per replicate. Root length and fresh weight were recorded prior to bolting, with drying procedures identical to the field experiment.

### 2.4 Stress treatment

To validate candidate genes, seeds of the CC2 line were sown in a mixed substrate of peat moss and vermiculite (1:1, v/v) and cultivated under controlled greenhouse conditions. At the five-true-leaf stage, seedlings were subjected to irrigation treatments as follows: (1) 100 g/L polyethylene glycol (PEG-6000) to simulate drought stress; (2) 100 μmol/L methyl jasmonate (MeJA) to simulate MeJA stress, according to established protocols (Sui et al., 2011; Yang et al., 2022). Control seedlings received distilled water. Root tissues were harvested at 0, 1, 2, 4, and 8 hours post-treatment initiation, flash-frozen in liquid nitrogen, and stored at −80°C for subsequent RNA extraction, saikosaponins quantification and gene expression validation. For each time point, seedlings from every three pots were pooled to constitute one biological replicate, yielding three biological replicates per treatment group.

### 2.5 Determination of saikosaponin content

Dried roots were ground to a fine powder through a 60-mesh sieve (Wanshi, China). For each sample, 0.5 g of root powder was extracted in 25 mL of 5% ammonia-methanol solution using ultrasonication for 30 min and then lyophilized. The dried extract was redissolved in 10 mL methanol. Saikosaponins A and D were quantified using a HPLC system (Waters, USA) equipped with an ASB-vensil C18 column (4.6 × 250 mm, 5 μm). Reference standards were obtained from the National Institutes for Food and Drug Control (Beijing, China), and analysis was performed following the method described by Xu (2019).

### 2.6 RNA-seq library preparation and sequencing

Fresh root samples of CBC, F_1_, and CC2 were collected in October 2021 for maturity and in October 2024 for seedlings, with three biological replicates for each sample. The roots were immediately frozen in liquid nitrogen and kept at -80°C for RNA extraction. Then, the samples were sent to Novogene Bioinformatics Technology Corporation (Beijing, China) for RNA-sequencing. The library construction and sequencing process have been reported by Yu et al. (2021).

### 2.7 RNA-seq data analysis and gene function annotation

The raw sequencing data were filtered by FastQC (Andrews, 2023) and Trimmomatic (Bolger et al., 2014). Clean reads were mapped to the CC2 genome (https://ym-lab.vip.cpolar.cn) using HISAT2 for further analysis (Kim et al., 2019). The reads count of each gene and original expression matrix were generated by featureCounts software (Liao et al., 2014). The matrix was then normalized by edgeR (Robinson et al., 2010) package in R (v. 4.2.2). The principal component analysis (PCA) and Pearson correlation coefficient analysis were conducted using the PCAtools in R. DESeq2 (Love et al., 2014) was used to identify differential expression genes (DEGs) among the two parents and F_1_ generation. Significant DEGs were filtered using a false-discovery rate (FDR) threshold of < 0.01 and ­log2 (FoldChange) > 1. Venn diagram analysis between samples was conducted using the TBtools (Chen et al., 2020). The software ClusterProfiler (Yu et al., 2012) was applied to perform KEGG enrichment analysis. The KEGG (Kanehisa et al., 2023) background was acquired from eggNOG-mapper v2 (Cantalapiedra et al., 2021). KEGG pathway analysis was applied to the “enricher()” function for enrichment. A hypergeometric test with a *P*-value threshold of 0.05 was applied.

### 2.8 Co-expression network analysis and hub gene identification

Weighted Gene Co-expression Network Analysis (WGCNA) was conducted with the R package WGCNA. The built-in function “goodSamplesGenes()” (with minFraction = 1/2) and the R package genefilter (with var.cutoff = 0.5) were applied to filter the normalized expression matrix. The filtered genes were used to construct a weighted co-expression network. The ME (module eigengene, the first principal component of a module) value was computed for each module to assess the relationship with the trait. The key genes were identified among the genes in the module most strongly associated with the trait, based on their connectivity (high kME values) and the number of network connections (more edges). The network of key genes was visualized using cytoscape 3.10 (Kohl et al., 2011). The top score hub genes were computed and sorted with the MCC method implemented in the Cytoscape plugin cytoHubba (Chin et al., 2014).

### 2.9 Candidate genes validation

Ten DEGs associated with root morphotype and saikosaponin biosynthesis were selected for qRT-PCR validation. Gene-specific primers were designed using Primer3Plus (Untergasser et al., 2012) with the following parameters: amplicon size = 80–150 bp, primer length = 18–25 nt, Tm = 58–62°C. Exon-spanning primers were employed to prevent genomic DNA amplification. Primer sequences are listed in **Supplementary Table S1**.

qRT-PCR amplification was performed using cDNA templates from: (1) stress-treated CC2 seedlings and (2) three parental genotypes used for RNA sequencing. Reactions utilized the TransStart Top Green qPCR SuperMix Kit (TransGen Biotech, China) on a LightCycler 96 system (Roche Diagnostics, Switzerland). The *BcADF5* gene (Yu et al., 2019) served as the endogenous reference. Gene expression levels were quantified using the 2^−ΔΔCt^ method (Livak and Schmittgen, 2001). Three biological replicates were analyzed, each with three technical replicates.

### 2.10 Statistical analysis

Analysis of variance (ANOVA) for all traits was performed using SPSS software v21.0 (SPSS Inc., Chicago, USA).

## 3 Results

### 3.1 Karyotype analysis

The root-related traits of F_1_ hybrids were better than those of both parental lines during both seedling (**Figure 1A**) and maturity (**Figure 1B**) stages. Through karyotype analysis, the parental line CBC (2n=2x_1_ = 12, x_1_ = 6) was found to possess 12 somatic chromosomes (**Figure 1C**), while CC2 (2n=4x_2_ = 20, x_2_ = 5) contained 20 chromosomes (**Figure 1D**). The F_1_ hybrids (2n=x_1_+2x_2_ = 16) exhibited 16 chromosomes (**Figure 1E**), half of the combined parental chromosome number. FISH with 18S rDNA probes revealed that both parental lines CBC and CC2 exhibited two fluorescent signals localized on homologous chromosomes, which were conserved in F_1_ hybrids. The 5S rDNA probe signals followed a similar inheritance pattern to chromosome numbers: CBC displayed two signals, CC2 showed four signals, and F_1_ hybrids carried three signals.

### 3.2 Phenotypic evaluation

**Figure 2:**
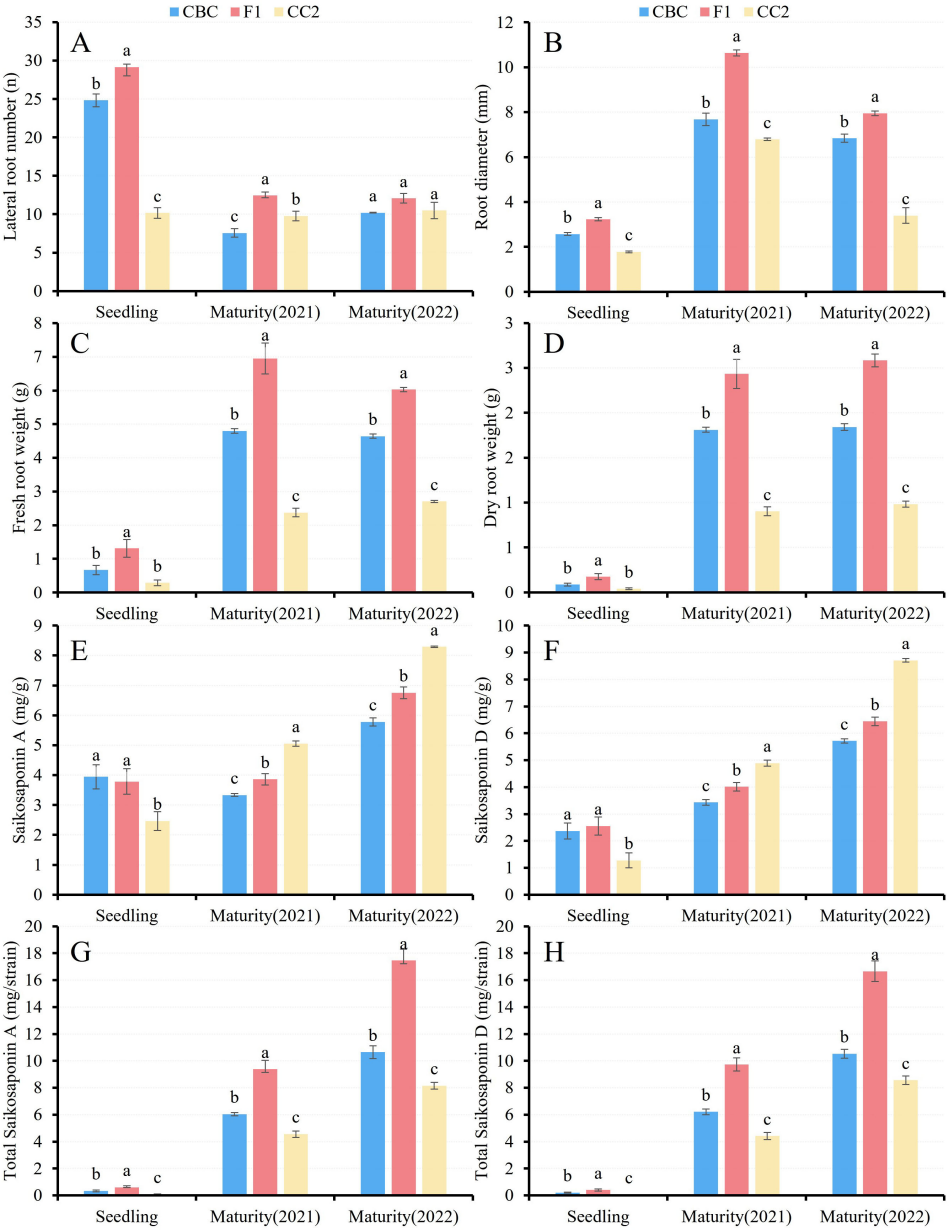
Statistical and correlation analysis of root-related traits and content of saikosaponin in F_1_ and parental lines. **(A)** Lateral root number; **(B)** Root diameter; **(C, D)** Fresh root weight and dry root weight; **(E, F)** saikosaponin A and D content; **(G, H)** Average yield of saikosaponin **(A, D)** per plant; The bar represents the standard error (SE). Different letters represent significant difference (*P* < 0.05).

At the seedling stage, the F_1_ hybrids displayed significant over-dominance (*P* < 0.05) in lateral root number, root diameter, root fresh weight, root dry weight, content of saikosaponin A and D, and the yields of saikosaponin A and D (**Figure 2**; **Supplementary Table S2**). For instance, F_1_ hybrid developed 29 lateral roots, exceeding CBC and CC2 by 1.2-fold and 1.5-fold, respectively; root dry biomass (0.17 g) exceeded CBC and CC2 by 89% and 325%, respectively; while saikosaponin A yield ranked F_1_ (0.61 mg) > CBC (0.34 mg) > CC2 (0.10 mg), with saikosaponin D following an identical trend.

Two-year field trails confirmed F_1_ hybrids over-dominance in root architecture (lateral root number, diameter, fresh/dry weight) and saikosaponin yields (**Figures 2A-H**; **Supplementary Table S2**). Mean root dry weight reached 2.50 g in F_1_, exceeding CBC (1.83 g) by 37.3% and CC2 (0.94 g) by 166.0%. Saikosaponin yields averaged 13.42 mg/plant for saikosaponin A (60.7% higher than CBC; 111.2% higher than CC2) and 13.20 mg/plant for saikosaponin D (57.5% higher than CBC; 103.3% higher than CC2). Although Saikosaponins contents in F_1_ were lower than in CC2, they surpassed CBC by 16.3% (saikosaponin A) and 14.3% (saikosaponin D).

Correlation analysis (**Supplementary Figure S2**) revealed that lateral root number negatively correlated with root diameter (r_n-rd_= -0.52), dry weight (r_n-dw_ = -0.48), and fresh weight (r_n-fw_ = -0.43). Extreme collinearity occurred between dry and fresh weights (r_dw-fw_ = 0.99), followed by root diameter and dry/fresh weight (r = 0.97). Saikosaponin A and D metrics showed positive associations with all root size parameters.

### 3.3 Transcriptome profile analysis

**Figure 3:**
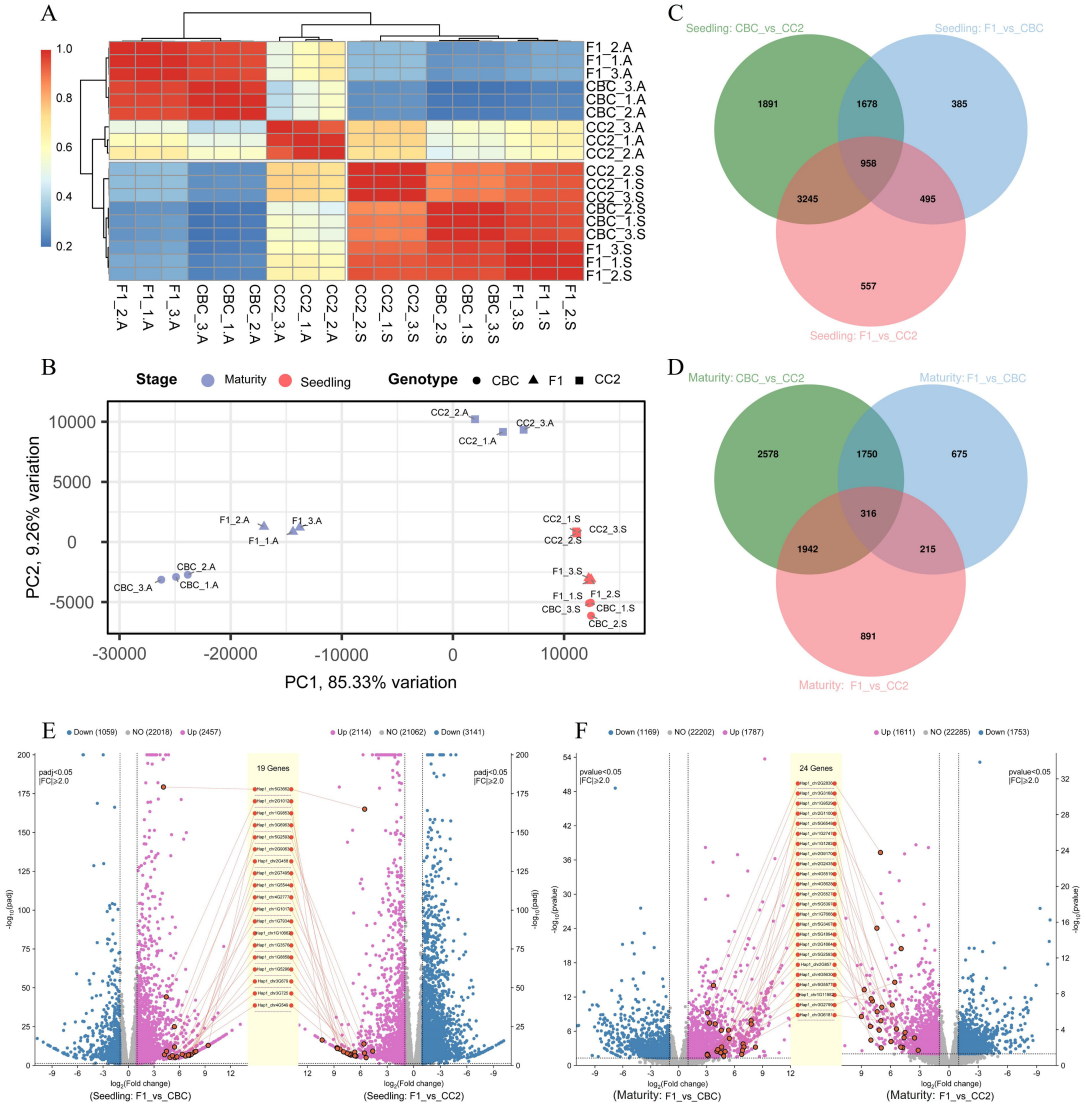
Bioinformatics analysis of transcriptome differential genes between F_1_ and parental lines. **(A)** Dendrogram heat map; **(B)** Principal component analysis; **(C)** Venn diagram of differential genes in seedling stage; **(D)** Venn diagram of differential genes in maturity stage; **(E)** Double volcano map between F_1_ and parental lines at seedling stage; **(F)** Double volcano map between F_1_ and parental lines at maturity stage.

Transcriptome sequencing of 18 biological samples (2 stages × 3 genotypes × 3 replicates) generated 54.18 Gb clean data. All libraries exceeded quality thresholds (Q30 > 94.29%, mapping rate > 80.0%), with high inter-replicate correlations (r>0.95) (**Figure 3A**; **Supplementary Table S3**). PCA showed dominant stage and genotype separation (PC_1_ = 85.33% variance; PC_2_ = 9.26% variance) (**Figure 3B**).

Differential expression analysis revealed genotype-distinct transcriptional profile, with seedling-stage (**Figure 3C**; **Supplementary Figure S3A**) comparisons identifying 7,772 DEGs between CBC and CC2 (2,771 up-/5,001 down-regulated), 3,516 DEGs between F_1_ and CBC (2,457 up-/1,059 down-regulated), and 5,255 DEGs between F_1_ and CC2 (2,114 up-/3,141 down-regulated). At the maturity stage (**Figure 3D**; **Supplementary Figure S3B**), DEG counts decreased to 6,586 for CBC *vs* CC2 (2,771 up-/3,815 down-regulated), 2,956 for F_1_*vs* CBC (1,787 up-/1,169 down-regulated), and 3,364 for F_1_*vs* CC2 (1,611 up-/1,753 down-regulated). Volcano plots detailed F_1_-specific expression patterns: 19 genes were upregulated in F_1_ versus both parents at the seedling stage (**Figure 3E**), increasing to 24 genes in maturity (**Figure 3F**).

Transcriptomic shock classification (Rapp et al., 2009) showed stage-dependent divergence (**Table 1**): maternal-dominant expression prevailed at both stages (seeding with 2,168 DEGs; maturity with 1,623 DEGs), followed by paternal-dominance. While additive patterns decreased 73.9% from seedling to maturity stages. Over-dominant genes declined by 58.3%, from 242 DEGs at seedling stage to 101 DEGs at the maturity stage.

**Table 1:** Possible additive and nonadditive gene expression patterns in a F1 hybrid relative to its parents.

Patterns	Stage	Additive expression		Parental lines expression				Transgressive downregulation			Transgressive upregulation			Total
Categories		I	XII	II	XI	IV	IX	III	VII	X	V	VI	VII	
Genotypes		♀ H ♂	♀ H ♂	♀ H ♂	♀ H ♂	♀ H ♂	♀ H ♂	♀ H ♂	♀ H ♂	♀ H ♂	♀ H ♂	♀ H ♂	♀ H ♂	
Expression levels		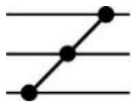	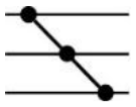	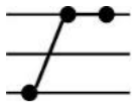	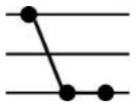	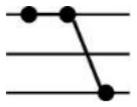	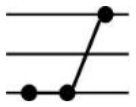	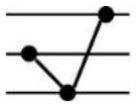	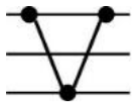	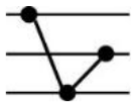	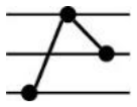	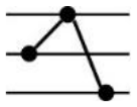	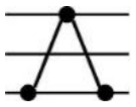
Genes number	Seedling	680	171	1089	223	1209	959	28	137	18	24	37	181	4756
	Maturity	177	103	1099	103	952	671	4	95	9	12	11	78	3314
Frequency (%)	Seedling	14.3	3.6	22.9	4.69	25.42	20.16	0.59	2.88	0.38	0.5	0.78	3.81	100
	Maturity	5.34	3.11	33.16	3.11	28.73	20.25	0.12	2.87	0.27	0.36	0.33	2.35	100

Roman numerals indicate the same categorization as used in Rapp et al. (2009) with figures schematizing their respective gene expression pattern for CBC as maternal parent (♀), F_1_ as hybrid (H), and CC2 as paternal parent (♂). The difference between each Expression level pvalue < 0.05.

The KEGG pathway analysis identified 17 significantly enriched metabolic pathways (*P* < 0.05) at the seedling stage and 21 at the maturity stage, with 11 core pathways conserved across both developmental stages including phenylpropanoid biosynthesis, sesquiterpenoid and triterpenoid biosynthesis, zeatin biosynthesis, and brassinosteroid biosynthesis (**Supplementary Figure S4**). Phenylpropanoid biosynthesis exhibited the highest DEG enrichment, displaying 115 and 79 DEGs in CBC *vs*. CC2 comparisons at seedling and maturity stages respectively, while F_1_*vs*. CBC comparisons revealed 47 (seedling) and 32 (maturity) DEGs. Notably, F_1_*vs*. CC2 comparisons showed 79 DEGs at the seedling stage but an absence of DEGs at maturity.

### 3.4 WGCNA and hub gene identification

**Figure 4:**
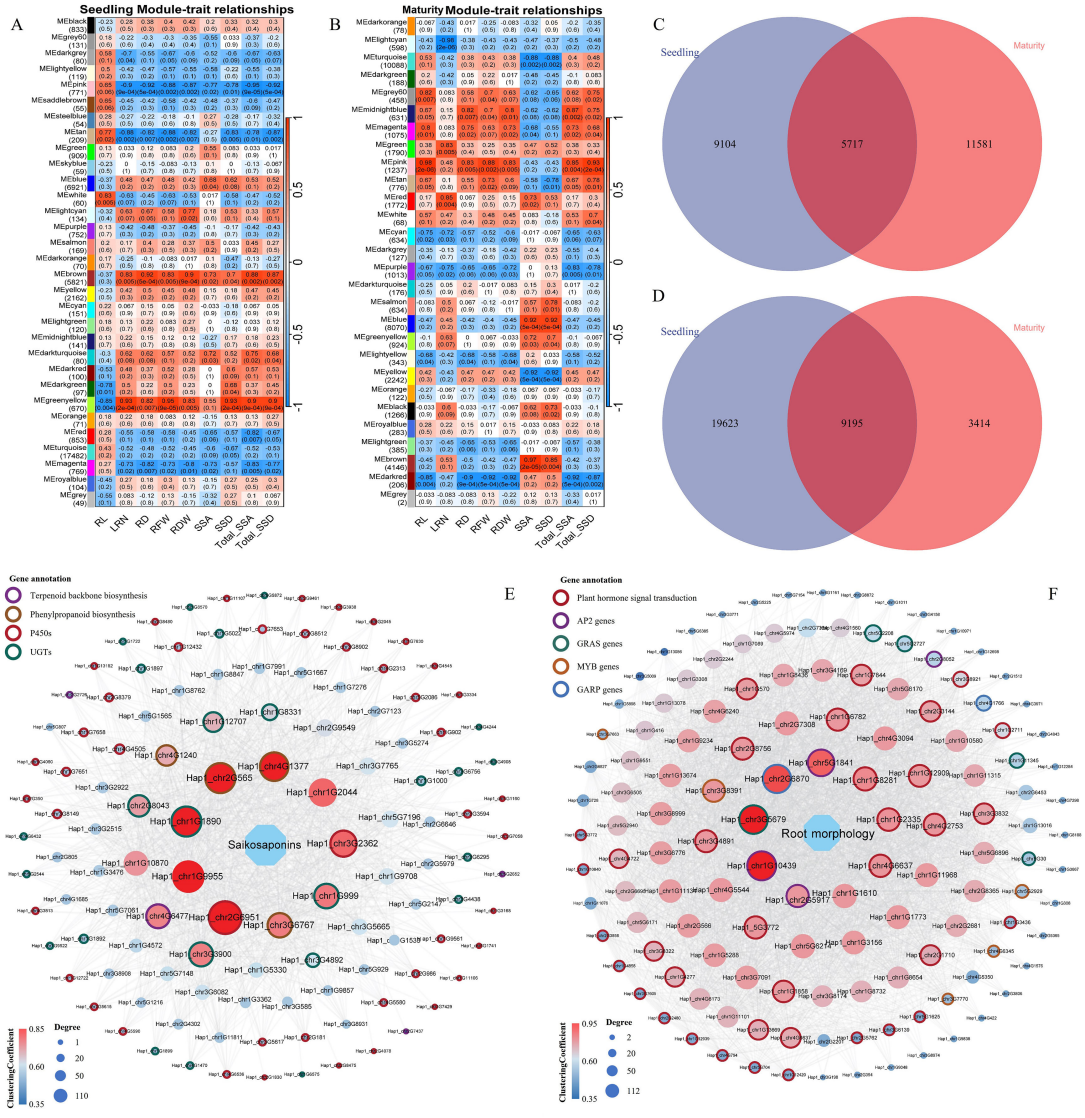
Weighted gene co-expression network analysis (WGCNA) of root genes at seedling and maturity stages. **(A)** Correlation analysis of gene modules and phenotypic traits at seedling stage; **(B)** Correlation analysis of gene modules and phenotypic traits at maturity stage. The modules on the left are color-coded, with the number of genes under the module name. Heatmap showing correlations between co-expression modules (vertical axis) and traits (horizontal axis). *P*-values are shown inside parentheses; **(C)** Venn diagram of genes related to saikosaponins content (SSA, SSD, Total_SSA and Total_SSD; module cor > 0.5) in the co-expressed gene modules at seedling and maturity; **(D)** Venn diagram of genes related to root morphology (RL, LRN, RD, RFW and RDW; module cor > 0.5) in the co-expressed gene modules at seedling and maturity; **(E, F)** Co-expression network of Saikosaponins and root morphology related modules; The size of nodes represents the Degree value of genes; The color of nodes represents the ClosenessCentrality value; and the color of the outer ring of nodes represents gene function or family classification.

WGCNA identified 31 trait-associated modules from 39,996 seedling-stage genes (**Figure 4A**; **Supplementary Figure S5A**). Lateral root number, root diameter, biomass, and saikosaponin-related traits exhibited significant positive correlations (*P* < 0.05) with the MEbrown module (5,821 genes) and MEgreenyellow module (670 genes) and the correlation coefficient were above 0.73, while showing negative correlations (*P* < 0.01) with the MEpink module and the correlation coefficient was less than -0.7. Root length demonstrated exclusive positive correlations with MEwhite and MEtan modules (*P* < 0.05). Analysis of 39,332 maturity-stage genes revealed 28 modules (**Figure 4B**; **Supplementary Figure S5B**). Root architecture traits and saikosaponin yields correlated positively with MEpink, MEmagenta, and MEMidnightblue modules (r > 0.83, *P* < 0.05), but negatively with MEdarkred module (r = -0.87, *P* < 0.01). Lateral root number only showed positive associations with MEred (1,772 genes) and MEgreen modules (1,790 genes).

Comparative analysis of phenotype-associated modules across developmental stages identified 5,717 conserved genes in saikosaponin-related modules (**Figure 4C**) and 9,195 in root morphology (**Figure 4D**) modules, prompting construction of dedicated co-expression networks. The saikosaponin biosynthesis network (**Figure 4E**) exhibited significant enrichment in specialized metabolic pathways including phenylpropanoid biosynthesis (ko00940), terpenoid backbone biosynthesis (ko00900), and saikosaponin-modifying enzymes—particularly cytochrome P450s and UGTs. Among its top 10 hub genes, functional annotation revealed two P450s (*Hap1_chr2G6951*, *Hap1_chr3G2362*), three UGTs (*Hap1_chr1G1890*, *Hap1_chr1G999*, *Hap1_chr3G3900*), three phenylpropanoid pathway genes (*Hap1_chr2G565*, *Hap1_chr4G1377*, *Hap1_chr3G6767*), and two transcription factors (WRKY family member *Hap1_chr1G9955* and PHD-finger protein *Hap1_chr1G2044*). While, the root architecture network (**Figure 4F**) showed pronounced enrichment in plant hormone signal transduction (ko04075), with core hubs dominated by transcription factor families: MYB proteins, AP2/ERF regulators, and GRAS members. Its top 10 hub genes comprised four hormone signaling components, three AP2 factors, one GRAS transcription factor (*Hap1_chr3G5679*), and one GARP family regulator.

### 3.5 Key genes associated with root morphology

**Figure 5:**
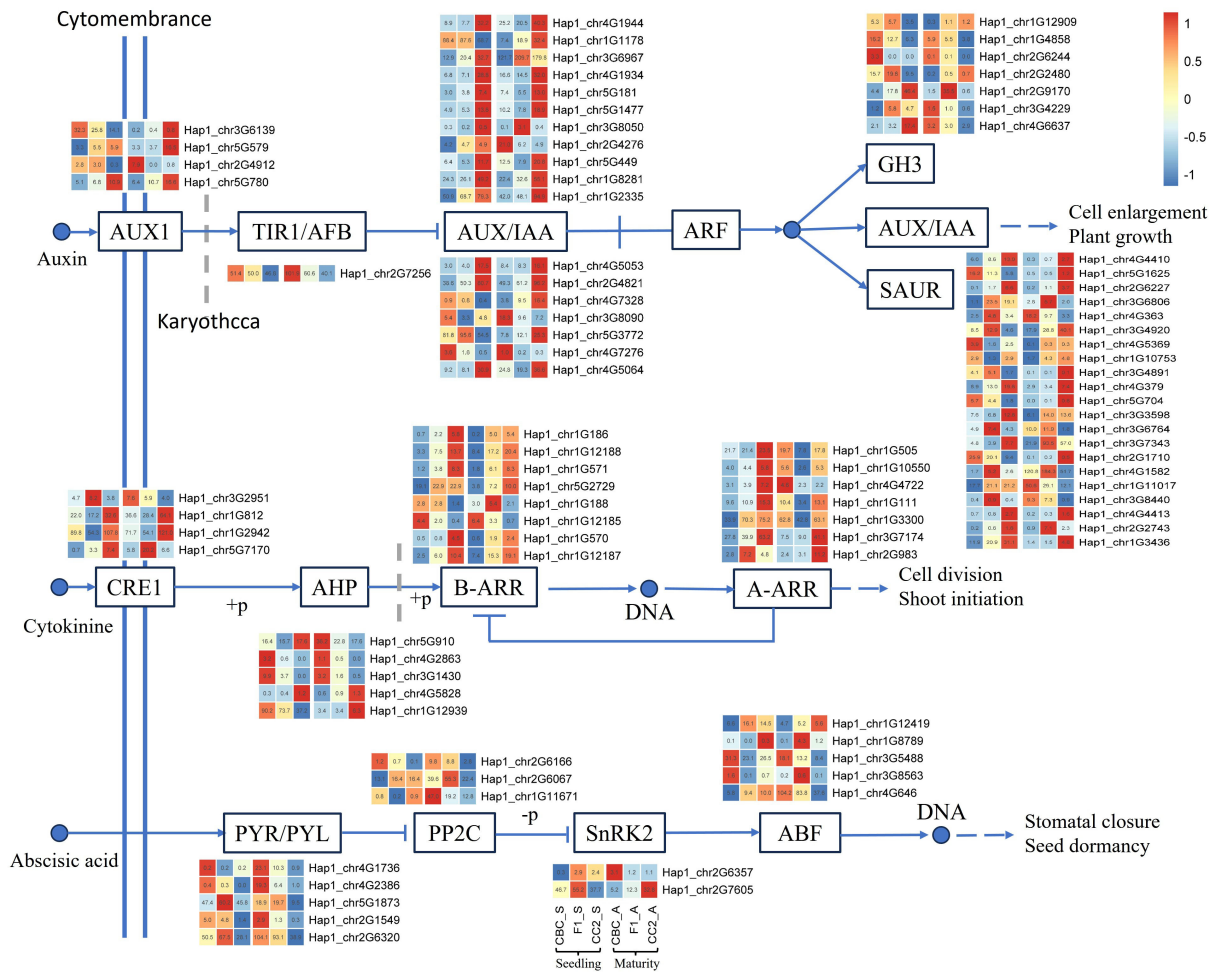
The core differentially expressed genes (Log2Foldchange > 1) from WGCNA analysis associated with root related traits in F_1_ and parental lines. In the heat map, the three columns on the left are seedling samples (CBC, F_1_, CC2 in order), and the three columns on the right are maturitysamples (CBC, F_1_, CC2 in order), and the heat map data of seedling and maturitysamples are normalized in “row” respectively.

Integrated WGCNA and differential gene analysis revealed 147 root morphology-associated DEGs enriched in plant hormone signaling pathways (**Figure 5**; **Supplementary Figure S6**), distributed as follows: 51 DEGs in the auxin (Aux) pathway, 24 in the cytokinin (CTK) pathway, 11 in the gibberellin (GA) pathway, 15 in the abscisic acid (ABA) pathway, 9 in the ethylene (ETH) pathway, 14 in the brassinosteroid (BR) pathway, 15 in the jasmonic acid (JA) pathway, and 8 in the salicylic acid (SA) pathway.

Within the auxin signaling pathway, the AUX/IAA gene family comprised 18 differentially expressed genes (DEGs), the GH3 family contained 8 DEGs, and the SAUR family included 21 DEGs. Notably, *BcIAA13* (*Hap1_chr1G8281*) exhibited significant downregulation in parental comparisons (CBC *vs*. CC2) across both developmental stages, while demonstrating additive expression in F_1_ hybrids (**Table 1**).

Within the cytokinin signaling pathway, eight GARP-family ARR-B genes and seven ARR-A transcriptional regulators were identified. The gibberellin pathway contained eight GRAS-family DELLA genes, while ethylene signaling featured eight AP2-family AP2-ERF genes. Notably, two ethylene-responsive ERF genes (*Hap1_chr1G4638* and *Hap1_chr2G1753*) exhibited transgressive upregulation (**Table 1**) in F_1_ hybrids across both developmental stages—a pattern mirrored in brassinosteroid signaling by TCH4 enzymes *Hap1_chr1G9738* and *Hap1_chr2G8015*. In jasmonic acid pathways, ten bHLH-family MYC2 genes were detected, including *Hap1_chr2G8944* with significantly elevated expression in CC2 (*P_adj_* < 0.05; 4-fold > F_1_, 7-fold > CBC). This gene displayed additive expression in seedlings and maternal-biased low expression in maturity (**Table 1**). Salicylic acid pathways contained four bZIP-family TGA genes, while abscisic acid signaling featured five bZIP-family ABF regulators.

### 3.6 Genes associated with saikosaponins synthesis

**Figure 6:**
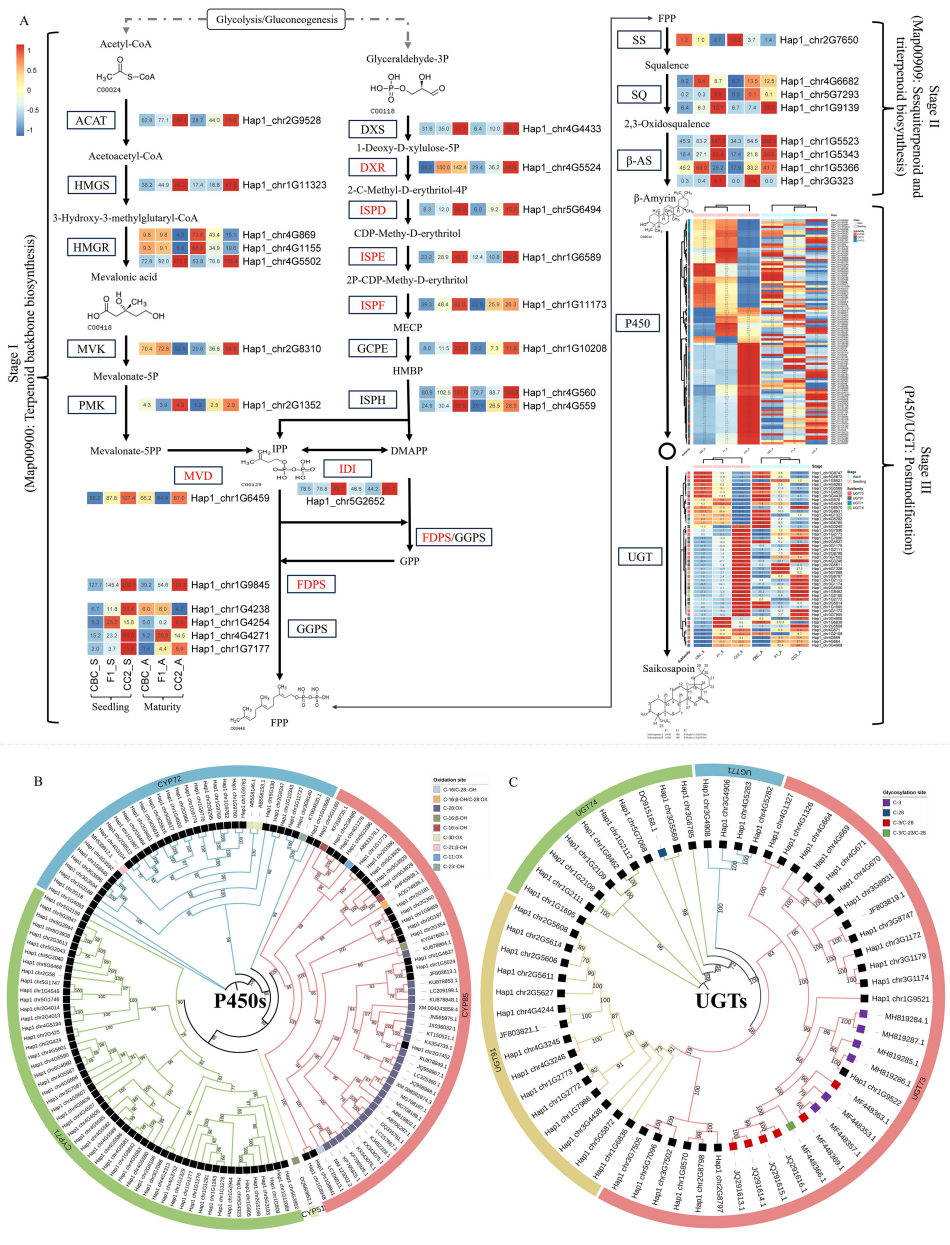
The core differentially expressed genes (Log2Foldchange > 1) from WGCNA analysis associated with saikosaponins content in F_1_ and parental lines. **(A)** In the heat map, the three columns on the left are seedling samples (CBC, F_1_, CC2 in order), and the three columns on the right are maturity samples (CBC, F_1_, CC2 in order), and the heat map data of seedling and maturity samples are normalized in “row” respectively; **(B, C)** Evolutionary relationship of differential expressed genes in hub Cytochromes P450 (P450s) and UDP-glucuronosyltransferases (UGTs) gene families, respectively. The number on the branch of the evolutionary tree represents the bootstrap value.

Integrated WGCNA-KEGG analysis revealed 179 DEGs enriched in saikosaponin biosynthesis, spanning three pathway stages. (**Figure 6A**). Terpenoid backbone biosynthesis (ko00900) contained 21 enzyme-encoding DEGs, including three HMGR genes (*Hap1_chr4G869*, *Hap1_chr4G1155*, *Hap1_chr4G5502*) - key rate-limiting enzymes in the MVA pathway - and a single-copy DXR gene (*Hap1_chr4G5524*) from the MEP pathway that showed significant inter-parental expression divergence (*P* < 0.01). Sesquiterpenoid/triterpenoid biosynthesis (ko00909) featured eight DEGs: one squalene synthase (SS, *Hap1_chr2G7650*), three squalene epoxidases (SQE, *Hap1_chr4G6682*, *Hap1_chr5G7293*, *Hap1_chr1G9139*), and four saponin synthases (*β*-AS, *Hap1_chr1G5523*, *Hap1_chr1G5343*, *Hap1_chr1G5366*, *Hap1_chr3G323*). Post-modification stages comprised 148 DEGs, including 102 DEGs encoding CYP450s enzymes and 46 DEGs encoding UGTs (UDP-glycosyltransferases). The P450 genes were classified into three subfamilies: CYP71 (59 genes), CYP72 (30 genes), and CYP85 (13 genes). While, UGT genes were categorized into four subfamilies: UGT71 (four genes), UGT73 (22 genes), UGT74 (nine genes), and UGT91 (11 genes).

Phylogenetic relationships between DEGs encoding P450s and UGTs and functionally characterized catalytic genes were analyzed (**Figure 6B**). In the P450s phylogenetic tree, *Hap1_chr3G7452* clustered most closely with KU878849.1, which encodes a C-28 oxidase. Three tandemly duplicated genes (*Hap1_chr5G3928*, *Hap1_chr5G3929*, and *Hap1_chr5G3926*) formed a clade with AHF45909.1 (100% support), a known C-16α hydroxylase. Gene *Hap1_chr1G5524* segregated on a distinct branch with Bupleurum-derived JF803813, a gene of unknown function. In the UGTs phylogeny (**Figure 6C**), *Hap1_chr1G9522* showed closest affinity to C-3 glycosyltransferase MH819286.1, whereas *Hap1_chr3G5569* was phylogenetically proximate to C-28 glycosyltransferase *VhUGT74M1* (DQ915168.1. Additionally, *Hap1_chr4G4244* and *Hap1_chr3G8747* demonstrated the strongest homology to putative glycosyltransferases *BcUGT5* (JF803821.1) and *BcUGT3* (JF803819.1), respectively.

### 3.7 Candidate genes function verification

Candidate hub genes identified from transcriptome analysis were selected for further functional verification in *B. chinense.* These included two auxin response factors (*BcIAA13.1*, *BcSAUR24.1*), two cytochrome P450 genes (*BcCYP716Y1.1*, *BcCYP716A83.1*), and two glycosyltransferase genes (*BcUGT73.1*, *BcUGT74.1*).

**Figure 7:**
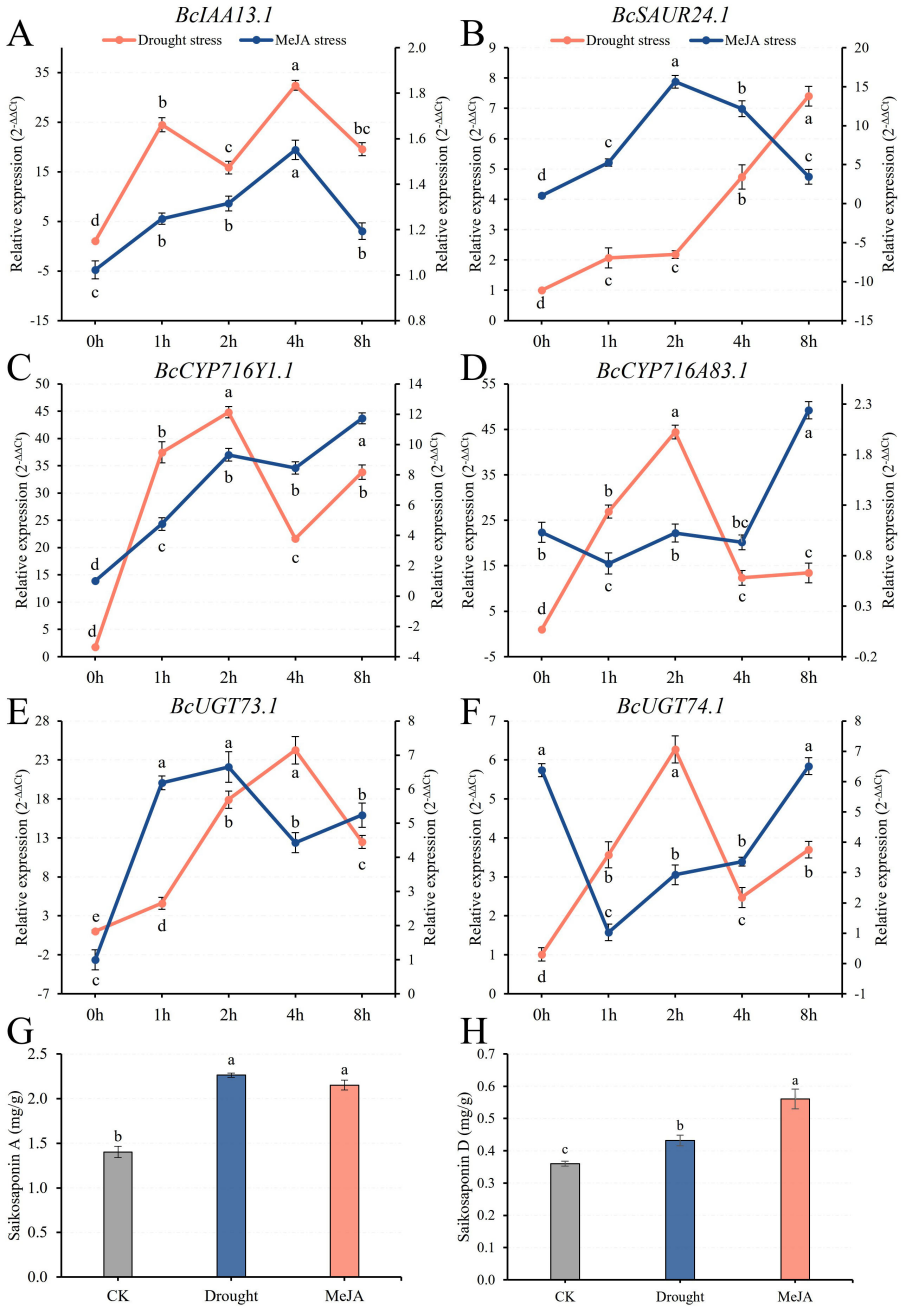
Candidate genes response and Saikosaponins accumulation under abiotic stress. **(A, B)** Auxin early response genes: *BcIAA13.1*, *BcSAUR24.1*; **(C, D)** Cytochromes P450: *BcCYP716Y1.1*, *BcCYP716A83.1*; **(E, F)** UDP-glucuronosyltransferases: *BcUGT73.1*, *BcUGT74.1*; **(G, H)** Saikosaponin A and D contents in Abiotic stress. Different letters represent statistically significant expression differences (P < 0.05).

Under drought stress, six candidate genes exhibited dynamic expression patterns across all measured time points (**Figures 7A-F**). *BcIAA13.1* and *BcSAUR24.1* were sustainedly up-regulated. In contrast, *BcCYP716A83.1* and *BcUGT74.1* expression peaked at 2 h. Gene *BcCYP716Y1.1* reached its maximum at 2 h, while *BcUGT73.1* showed a delayed peak at 4 h. Under MeJA treatment, the expression profiles differed: *BcIAA13.1* and *BcSAUR24.1* peaked at 4 h and 2 h, respectively. Genes *BcCYP716A83.1* and *BcUGT74.1* were initially down-regulated but subsequently stimulated, both reaching their highest expression at 8 h. Gene *BcCYP716Y1.1* was consistently up-regulated, peaking at 8 h, and *BcUGT73.1* expression peaked at 2 h before decreasing. The HPLC analysis results showed (**Figures 7G, H**) that the contents of saikosaponin A and D in *B. chinense* were significantly higher under drought and MeJA treatment compared to the control (CK). The effect of MeJA on the accumulation of saikosaponin D was significantly greater than that of drought stress (**Figure 7H**).

To further validate the transcriptome results, qRT-PCR was performed on seedling-stage samples of the F_1_ and parental lines using the six genes with differential expression identified under abiotic stress (**Supplementary Figure S7**). The expression trends obtained by qRT-PCR were highly consistent with the RNA-seq data, confirming the reliability of the transcriptome-derived differential expression profiles. In detail, *BcIAA13.1*, *BcCYP716Y1.1*, *BcUGT73.1* and *BcUGT74.1* displayed additive expression patterns, *BcCYP716A83.1* followed the parental expression pattern, and *BcSAUR24.1* exhibited transgressive up-regulation, all in agreement with the RNA-seq analysis (**Table 1**).

## 4 Discussion

This study developed novel triploid *B. chinense* germplasm through intraspecific hybridization, as summarized by a schematic model of heterosis mechanisms (**Supplementary Figure S8**): diploid CBC (Chuanbeichai #1, 2n=2x_1_ = 12, x_1_ = 6) was crossed as female parent with tetraploid CC2 (Chuanchai #2, 2n=4x_2_ = 20, x_2_ = 5) as male parent, yielding an F_1_ triploid population (2n=x_1_+2x_2_ = 16). The hybrids exhibited significant heterosis, with dry root yield reaching 1.37-fold that of CBC and total saikosaponin (A+D) content increasing to 1.59-fold, highlighting their pharmaceutical potential. Triploid plants which harbor three sets of chromosomes and exhibit significant advantages in biomass, metabolism, and stress tolerance are common in plants, particularly in in crops harvested for vegetative organs and seedless fruit crops (Wang et al., 2016; Ramakrishnan et al., 2025). For instance, watermelon ‘Vertigo’ (2n=3x=33) achieved yields up to 45.95 t/ha (Cushman et al., 2003). Cavendish banana (2n=3x=33) combined high productivity with strong resistance to *Fusarium* wilt (Li et al., 2024). Triploid dominance was also found in medicinal plants. Triploid *Siraitia grosvenorii* (2n=3x=42) enhanced yield and processing quality (Sreekumari et al., 1999) and produced fruits with 36.28% higher mogroside V content than diploids (Wei et al., 2022). Triploid *Eucommia ulmoides* (2n=3x=51) surpassed the diploids in growth rate, photosynthetic efficiency, and secondary metabolite accumulation (Li et al., 2019). *Salvia miltiorrhiza* triploids show 215.33% greater fresh root weight with tanshinone content exceeding Chinese Pharmacopoeia standards (Li and Chen, 2012). *Erigeron breviscapus* triploids produced 214% more biomass while maintaining 13% higher scutellarin content than diploids (Wu et al., 2011).

FISH revealed intergenomic triploid F_1_ hybrids derived from the CC2 (x=5) × CBC (x=6) cross, characterized by unequal basic chromosome numbers between parental genomes. This architecture differs fundamentally from conventional triploids and standard aneuploidy involving numerical variation within a single chromosome set (Zamariola et al., 2014). Typical triploids exhibit chromosome numbers exactly triple the haploid base (e.g., cassava 2n=3x=54, x=18; banana/watermelon 2n=3x=33, x=11) (Ramakrishnan et al., 2025). The observed intergenomic imbalance might also provide a stable genomic foundation for sustained heterosis. Chromosomal rearrangement between subgenomes could facilitate transcriptional reprogramming, enable modular partitioning of key biological pathways—such as root development and specialized metabolite biosynthesis—into complementary subgenomic units (Chen, 2013; Deb et al., 2023). This functional compartmentalization strategy might optimize existing parental pathways rather than relying on *de novo* evolution and show a sub-genome recombination case for optimizing perennial medicinal crops breeding.

Heterosis in *B. chinense* F_1_ hybrids was observed at both seedling and maturity stages, as revealed by integrated phenotypic and transcriptomic analyses. Since the root is the medicinal organ of this species, root biomass and the concentration of bioactive compounds determine its therapeutic quality. Two-year field trials and greenhouse experiments demonstrated significant heterotic advantages in root architecture traits including lateral root density, diameter, and biomass yield. Transcriptional upregulation underpins this heterosis, with parental-dominance expression patterns (> 70%) predominating among DEGs, and approximately 3% of DEGs in the F_1_ hybrids exhibited transgressive upregulation at both seedling and maturity stages (**Table 1**). This expression pattern diverged from rapeseed studies where transgressive regulation dominated F_1_ hybrids (Shalby et al., 2021) but aligned with rice research reporting prevalent paternal-dominant expression patterns. The triploid genomic constitution may further enhance metabolic capacity through gene dosage effects and allelic complementation between divergent parental genomes, whereby favorable alleles jointly optimize regulatory robustness and pathway efficiency (Chen, 2013; Schnable and Springer, 2013). In current study, significant heterosis manifested at both developmental stages, with greater DEG abundance observed during rapid seedling development. These findings suggested advantageous traits in mature plants might originate from developmental priming during early growth phases.

Root morphology-associated DEGs were enriched in plant hormone signaling transduction pathways. Auxin, as a key growth regulator, played a critical role in lateral root development. Within the auxin signal transduction pathway, the SCF^TIR1/AFB^-Aux/IAA-ARF module regulated the downstream genes expression. In this study, five genes in Aux/IAA family expressed relatively higher in parents than in the F_1_ generation. Through comparative transcriptome (Yu et al., 2021), *B. chinense* exhibited lower expression of the auxin inhibitor *BcIAA13* (*Hap1_chr1G8281*) but greater lateral root density than *B. scorzonerifolium*. This inverse correlation aligned with root suppression in rice *Osiaa13* gain-of-function mutants (Kitomi et al., 2012). The remaining four Aux/IAA genes were reported to inhibit lateral root initiation in different species. For instance, an auxin-resistant *Arabidopsis* mutant, *Atiaa28-1*, exhibited severe defects in lateral root formation, and reduced maturity size and decreased apical dominance (Rogg et al., 2001). In addition, Segmental duplication might also influence root development across plant species (Fan et al., 2018). Transgenic apple plants overexpressing *MdIAA27T* exhibited enhanced tolerance to phosphorus deficiency, characterized by the development of longer and denser adventitious roots (Zhao et al., 2022). *Sl-IAA27* expression was up-regulated by the AM fungus and had a positive impact on AM colonization (Guillotin et al., 2017). Ectopic expression of the auxin-responsive gene *TrIAA27* in *Arabidopsis* has been shown to promote biomass accumulation and improve tolerance to both drought and salt stress (Iqbal et al., 2024). Two tandem *BcIAA27* duplication pairs were identified on chromosome 4 in this study. *BcIAA27.1* (*Hap1_chr4G1934*) and *BcIAA27.2* (*Hap1_chr4G1944*) shared 100% sequence identity with about 120 kb segmental duplication; while, *BcIAA27.3* (*Hap1_chr4G5053*) and *BcIAA27.4* (*Hap1_chr4G5064*) exhibit >98% similarity with about 95 kb duplication. In the downstream of the auxin transduction pathway, sixteen tandemly arranged SAUR genes on chromosome 4 were found, among which only *BcSAUR24.1* (*Hap1_chr4G363*) and *BcSAUR50.1* (*Hap1_chr4G379*) exhibited differential expression in the F_1_ generation. Together, the additive expression of *BcIAA13* in the hybrid might stabilize auxin responsiveness and prevent excessive inhibition of lateral root formation, while the tandemly duplicated *BcIAA27* and *BcSAUR* genes might provide a genomic basis for enhanced environmental adaptability—demonstrating how hormone-related transcriptomic reprogramming modulates both biomass heterosis and adaptive trait optimization in *B. chinense.*

In *B. chinense*, more than 100 types of saikosaponins were reported, among which saikosaponin A and saikosaponin D were the primary medicinal components. On the pentacyclic triterpenoid saponins biosynthesis pathway, the post-modification genes P450s and UGTs have been extensively studied in different species (Li et al., 2023). The protein of P450s could participate in the hydroxylation and oxidation at the C-11, C-16, C-21, C-23, and C-28 positions of triterpenoid saponins. Phylogenetic analysis revealed that differentially expressed P450s were mainly grouped into subfamilies CYP72, CYP71, and CYP85, respectively (**Figure 6B**). In the *Bupleurum* genus, only *CYP716Y1* (AHF45909.1) classified into CYP85, has been cloned from *B. falcatum* (Moses et al., 2014), which catalyzed the C-16α hydroxylation of triterpenes. In the current study, a tandem of *BcCYP716Y1.1* (*Hap1_chr5G3928*) and *BcCYP716Y1.2* (*Hap1_chr5G3929*) were clustered in this group, and showed a significant inter-parental expression difference (*P* < 0.01), suggesting its potential role as a key driver of metabolic innovation in saikosaponin profiles. Zhang et al. (2022) identified 266 P450s genes from the genome of *B. chinense*, the sequences of these genes were unpublished, it was unable to determine the homology of these genes with those in our experiment. Transcriptome analysis by Sui et al. (2011) identified a candidate P450 oxidation gene, *BcCYP716A41* (JF803813.1), which shares high sequence identity (>98%) with *Hap1_chr1G5524*, suggesting that they might represent the same gene. In addition, *BcCYP716A83.1* (*Hap1_chr3G7452*) might possess a similar function to *CaCYP716A83* (KU878849.1), as they were clustered within the same phylogenetic clade. Further investigation is warranted to elucidate the functions of the 30 differentially expressed P450 genes in the CYP72 subfamily and the 53 differentially expressed P450 genes in the CYP71 subfamily. As for UGTs genes, glycosylation mostly happened at C-3, C-23 and C-28 positions of saikosaponins. Phylogenetic analysis indicated that *Hap1_chr4G4244* shared high identity (>84%) with *BcUGT5* (JF803821.1), and *Hap1_chr3G8747* shared high identity (>89%) with *BcUGT3* (JF803819.1) (Sui et al., 2021), suggesting that these sequences might represent the same gene (**Figure 6B**). In ginsenosides, the glycosyltransferase gene *PzOAGT3* (MH819286) transferred glucuronic acid at C-3 positions of oleanolic acid to form oleanolic acid 3-O-β-glucuronide (Tang et al., 2019). This gene showed a high sequence similarity (>77%) to *BcUGT73.1* (*Hap1_chr1G9522*), suggesting potential functional similarities (**Figure 6C**). The gene *UGT74M1* (DQ915168) from *Saponaria vaccaria* forming a glucose ester at C-28 position during monodesmoside biosynthesis (Meesapyodsuk et al., 2007). This gene was closely associated with *BcUGT74.1* (*Hap1_chr3G5569*), indicating a potential role in catalyzing glycosylation at the C-28 position (**Figure 6C**). Collectively, members of CYP71/72 and UGT73/74 subfamilies emerged as dominant players in the saikosaponin modification stage, with *BcCYP716Y1.1* exemplifying a parental-expression-divergent gene that may underpin novel metabolite profiles in the hybrid. Such specialized metabolic rewiring, in concert with hormone-regulatory networks, likely contributes to the optimization of both medicinal quality and ecological adaptability in *B. chinense*.

In addition, the mechanisms enabling hybridization between parents with unequal basic chromosome numbers, the stability of heterosis-associated transcriptional reprogramming during further domestication, and the feasibility of large-scale seed production require further investigation. Future studies combining multi-environment trials and functional genomics approaches will be essential to evaluate the stability and application potential of triploid breeding in *B. chinense*.

## 5 Conclusions

This study reported the karyotype profiles of two dominant *B. chinense* cultivars (CBC: 2n=2x_1_ = 12, x_1_ = 6; CC2: 2n=4x_2_ = 20, x_2_ = 5) and their triploid F_1_ hybrids (2n=x_1_+2x_2_ = 16), revealing a unique ploidy-driven genomic architecture. Alongside demonstrating root biomass heterosis, the F_1_ hybrids achieved a breakthrough in saikosaponin biosynthesis efficiency. The remarkable heterosis might be underpinned by unique genomic architecture and sophisticated transcriptional reprogramming. Intergenomic imbalance established a stable foundation for heterosis by facilitating functional compartmentalization and synergistic interaction between the parental subgenomes. Transcriptome analysis revealed that the heterotic traits were arranged by a complex relationship of gene networks: root morphology was optimized through the additive and transgressive expression of hormone signaling genes, while the enhanced synthesis of saikosaponins is driven by the synergistic and divergent expression of key biosynthetic genes. This research might exceed conventional plant breeding by demonstrating engineered genomic asymmetry, specifically intergenomic triploid and could help to unlock superior and stable heterosis.

## Data Availability

The RNA-seq data that support the findings of this study have been deposited into CNSA with accession number CNP0008950.
