# Supplementary material for: Heterosis unveiled in root-related traits and saikosaponins content between triploid F1 hybrids and parental Bupleurum chinense DC
Source: Front Plant Sci. 2026 Feb 12;17:1736464. doi: 10.3389/fpls.2026.1736464 (PMC12935959; doi:10.3389/fpls.2026.1736464)
Supplement: Supplementary file 1 [file DataSheet1.docx]

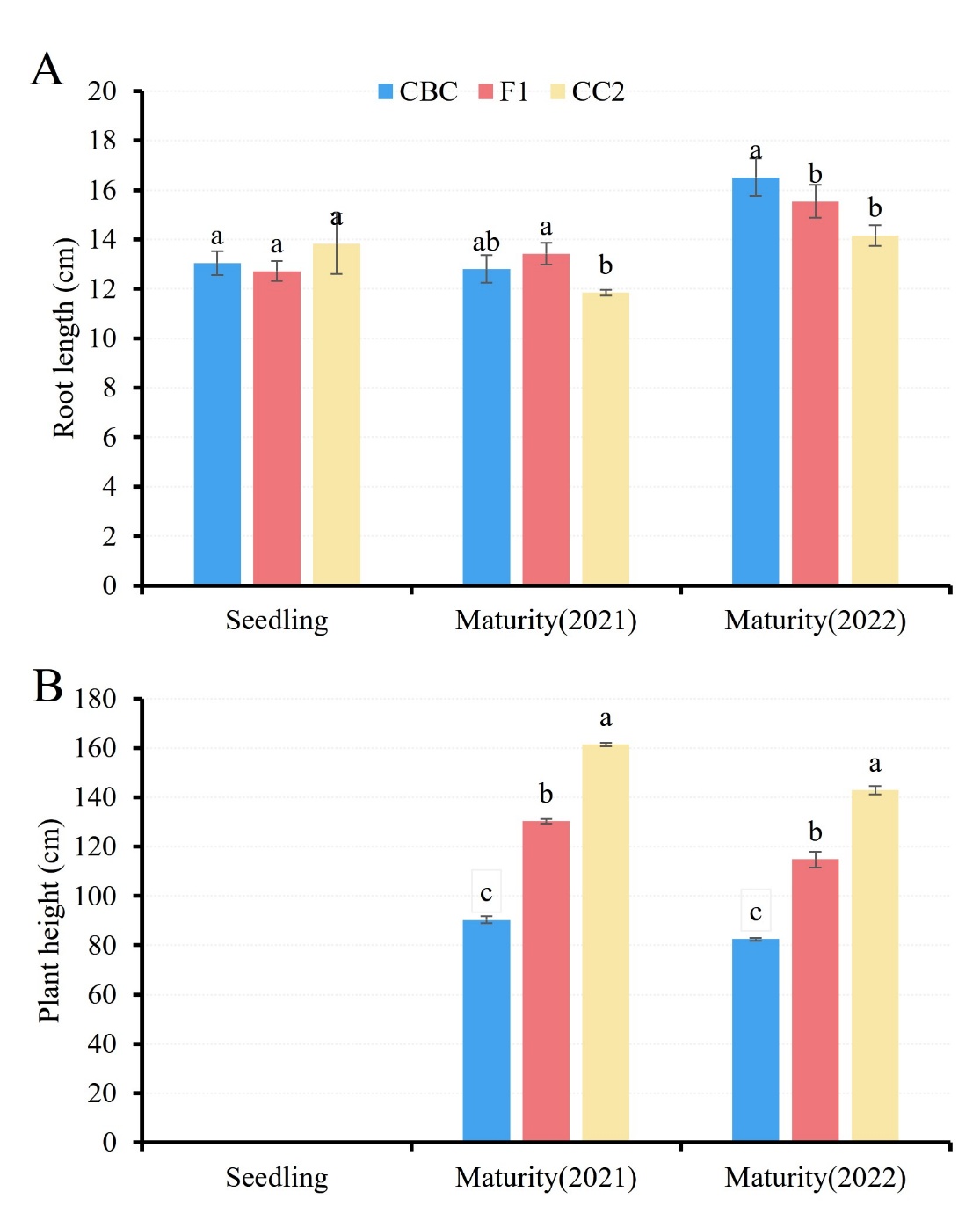


**FIGURE S1.** Statistical analysis of root length and plant height of F_1_ and its parents at seedling and maturity stages. (A) ANOVA of root length; (B) ANOVA of plant height; plant height data were not available at the seedling stage.


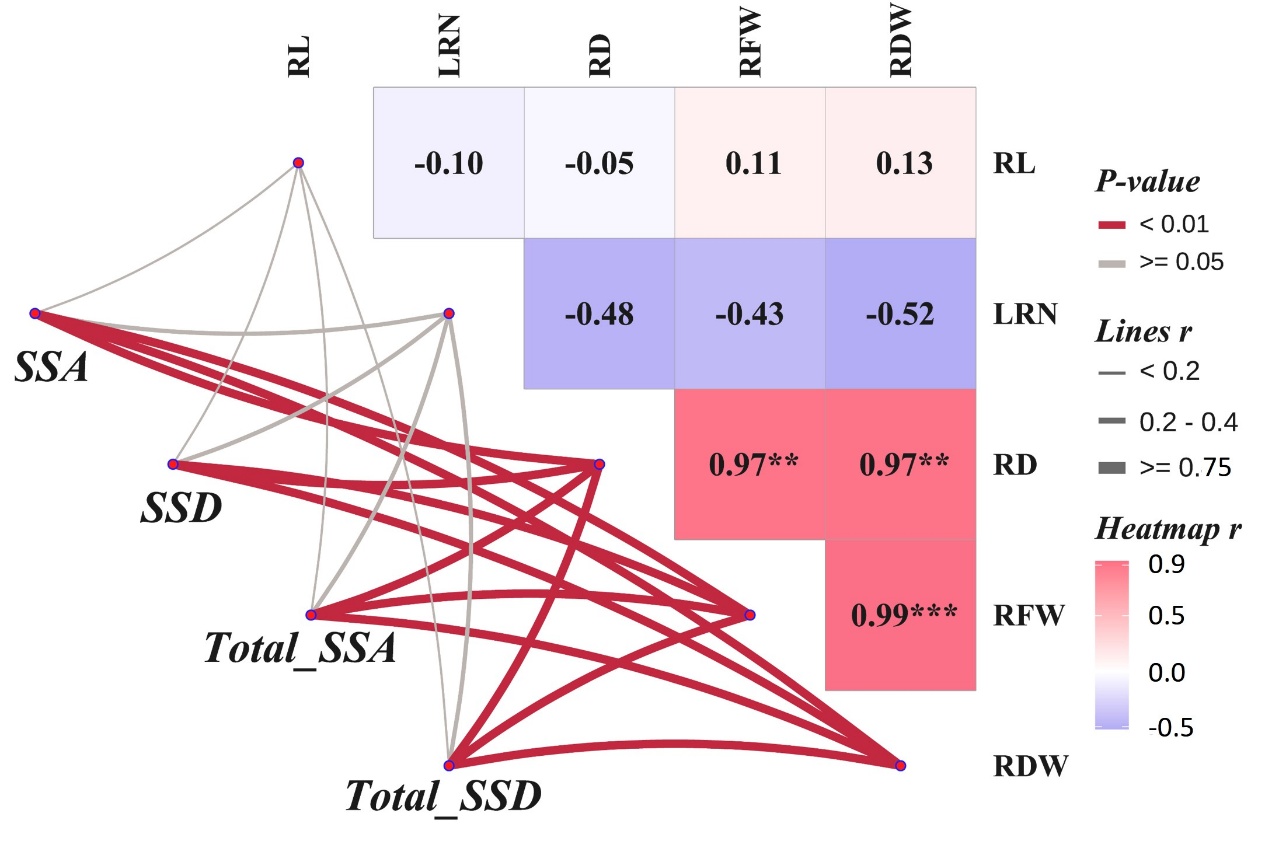


**FIGURE S2.** correlation between the content of Saikosaponins and phenotypes. (RL: root length; LRN: lateral root number; RD: root diameter; RFW: root fresh weight; RDW: root dry weight; SSA: saikosaponin A content; SSD: saikosaponin D content; Total_SSA: saikosaponin A yield; Total_SSD: saikosaponin D yield)


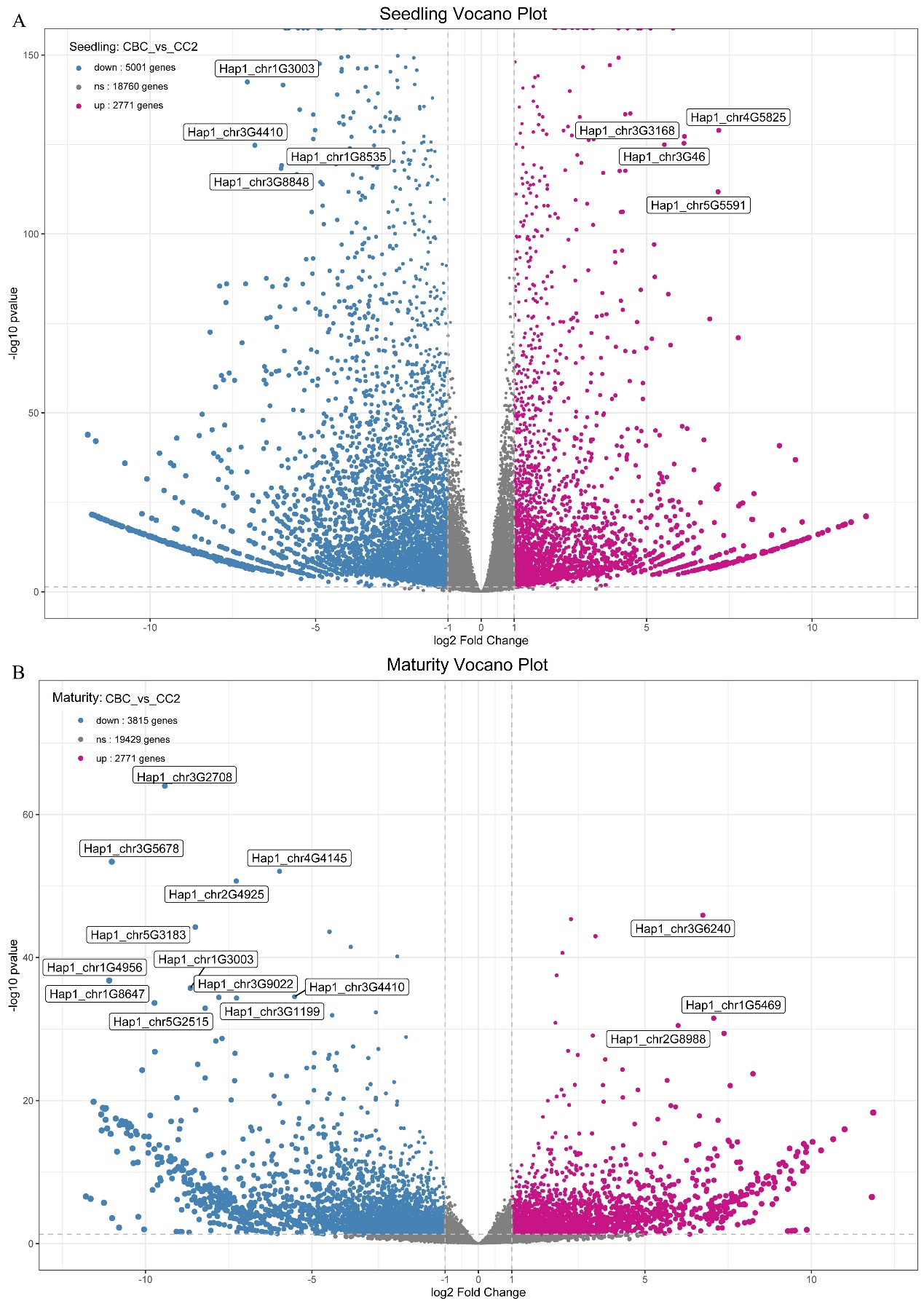


**FIGURE S3.** Differential gene volcano plot of parents CBC and CC2. (A) Seedling stage; (B) Maturity satge.


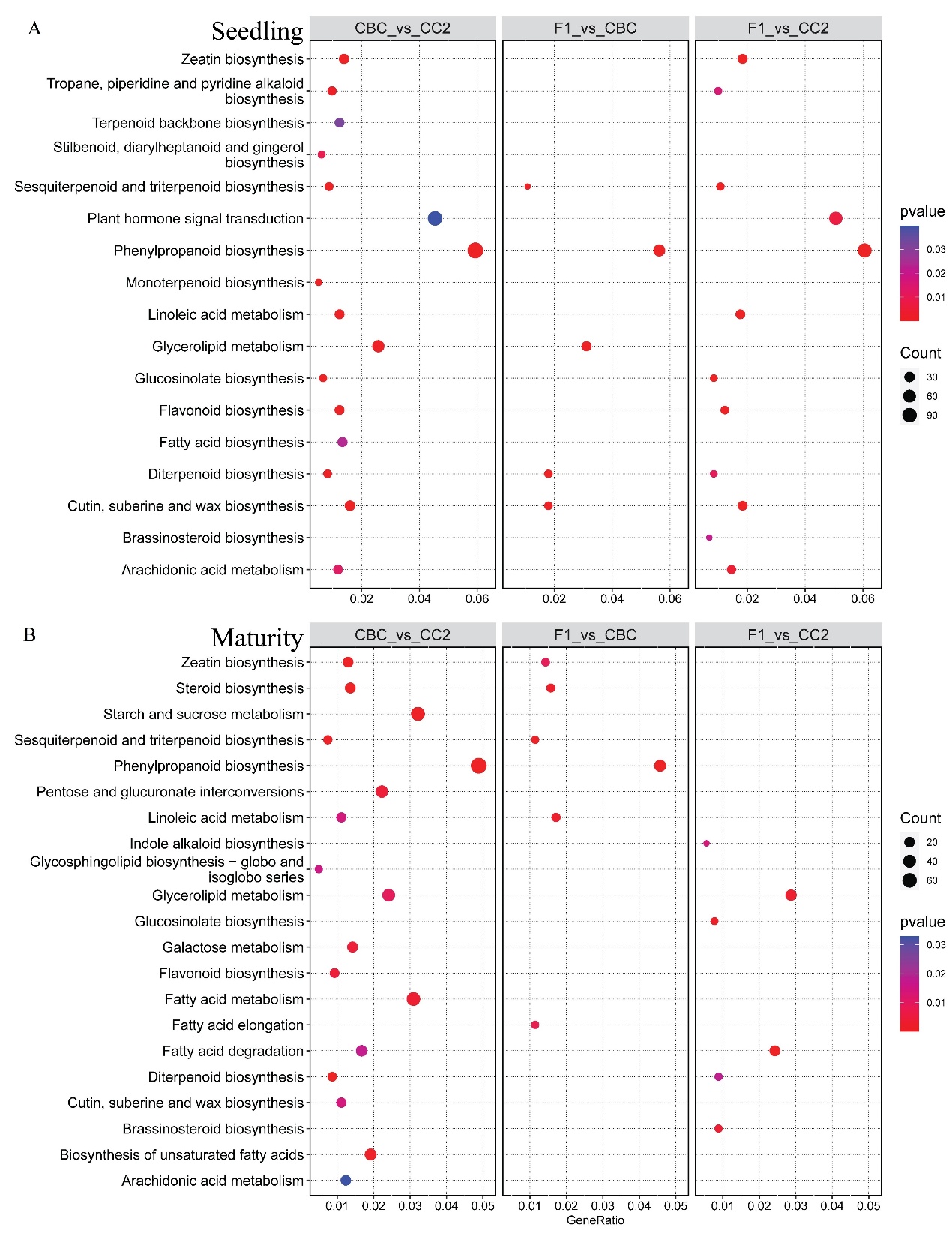


**FIGURE S4.** KEEG enrichment analysis of F1 and parental lines differential genes. (A) Seedling stage; (B) Maturity satge.


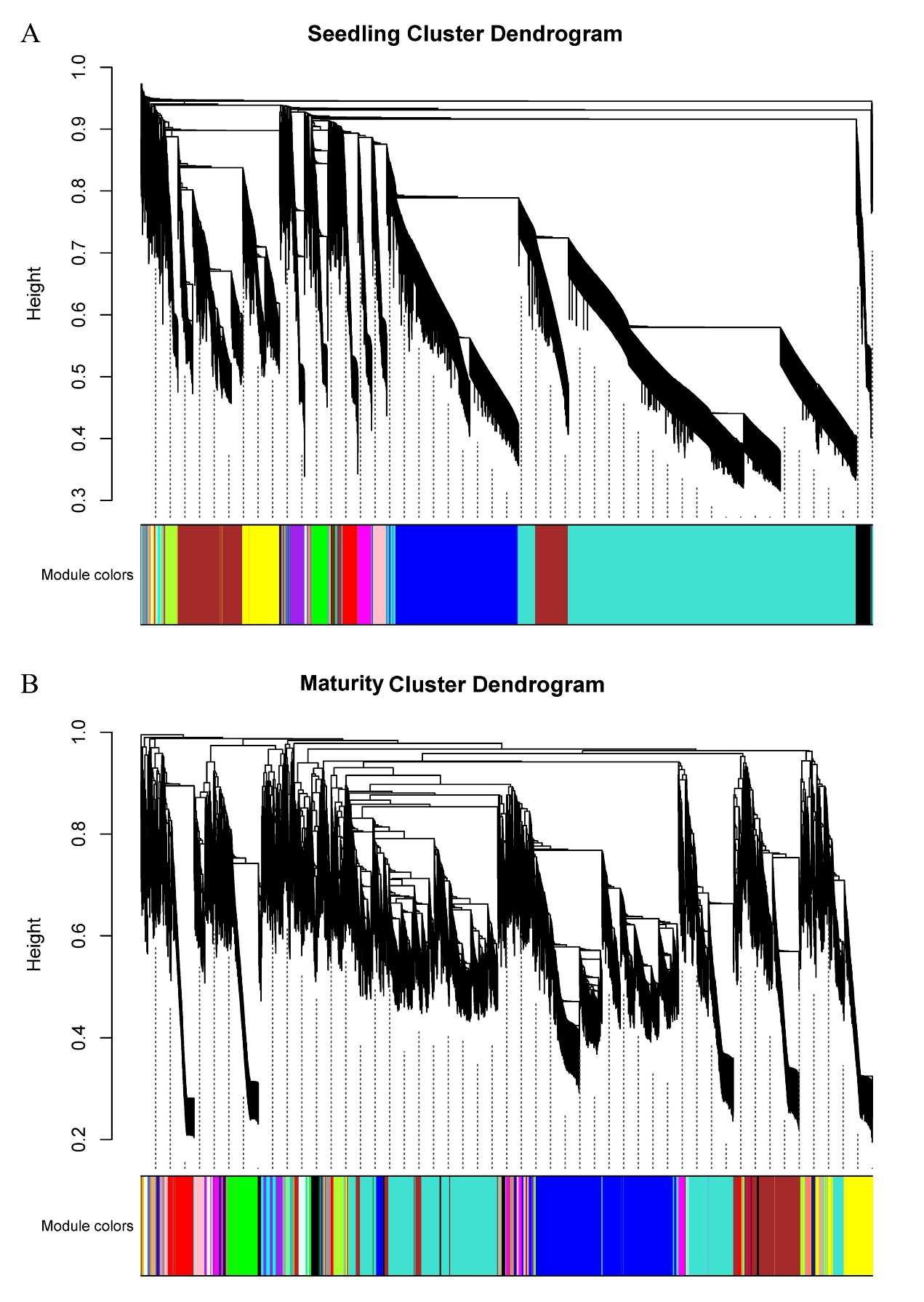


**FIGURE S5.** Hierarchical clustering tree of genes and co-expression modules. (A) Seedling stage; (B) Maturity stage.


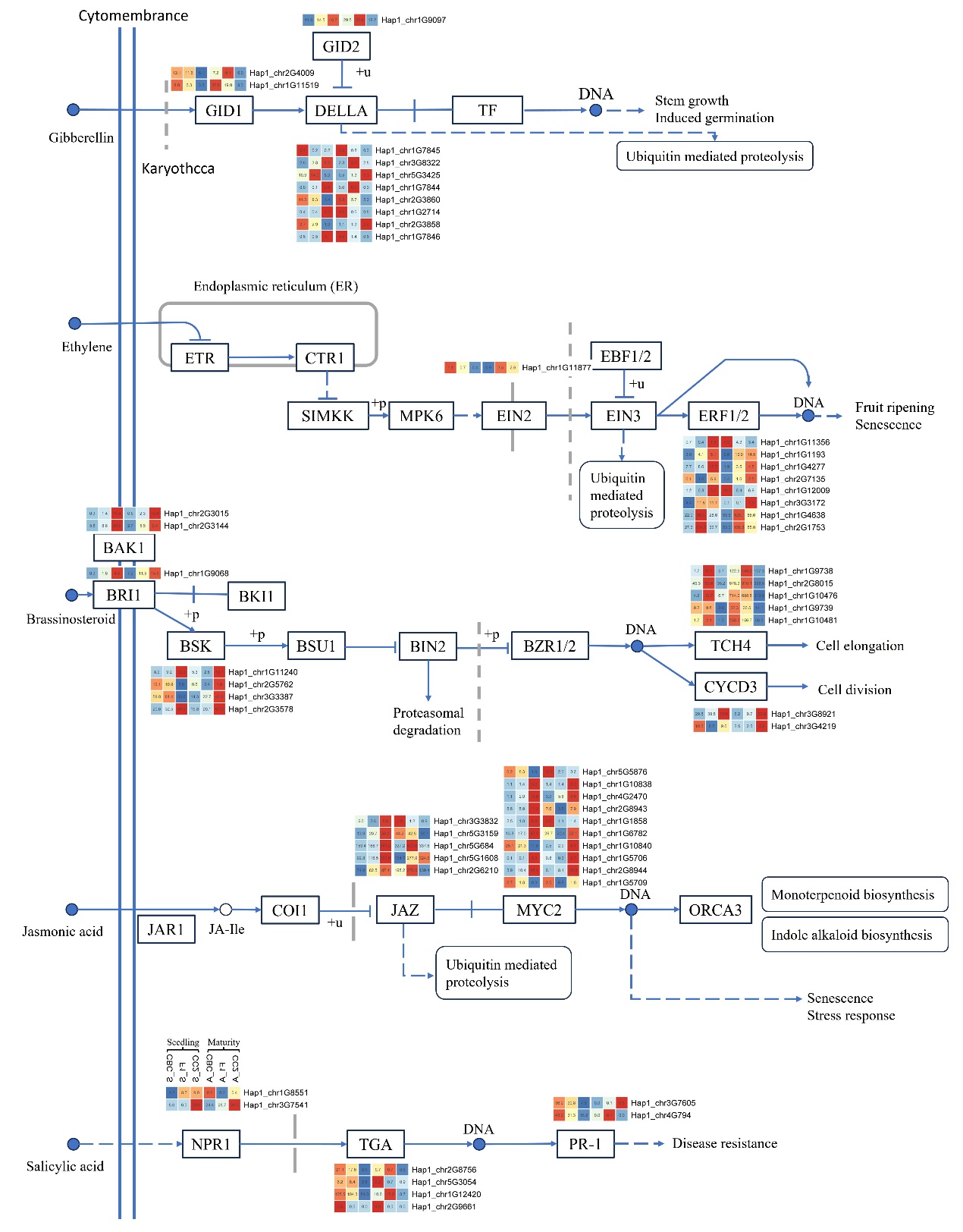


**FIGURE S****6.** Response patterns of key differential genes from WGCNA-related modules on plant hormone signal transduction pathways. In the heat map, the three columns on the left are seedling samples (CBC, F1, CC2 in order), and the three columns on the right are maturity samples (CBC, F1, CC2 in order), and the heat map data of seedling and maturity samples are normalized in "row" respectively.


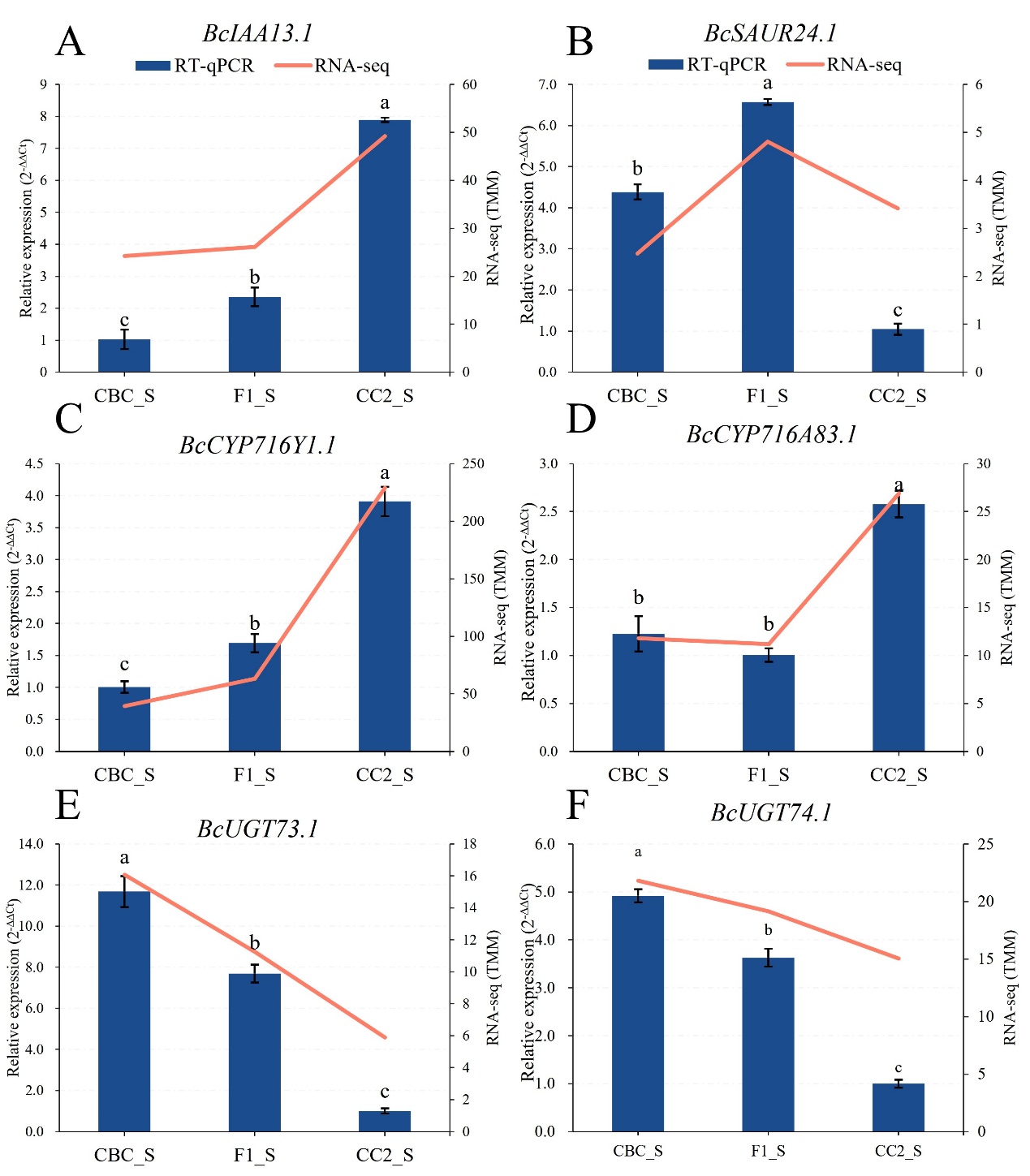


**FIGURE S7.** Validation of RNA-seq data of six candidate genes via qRT-PCR. (Auxin early response genes: *BcIAA13.1*, *BcSAUR24.1*; Cytochromes P450: *BcCYP716Y1.1*, *BcCYP716A83.1*; UDP-glucuronosyltransferases: *BcUGT73.1*, *BcUGT74.1*). Different letters represent statistically significant expression differences (P < 0.05).


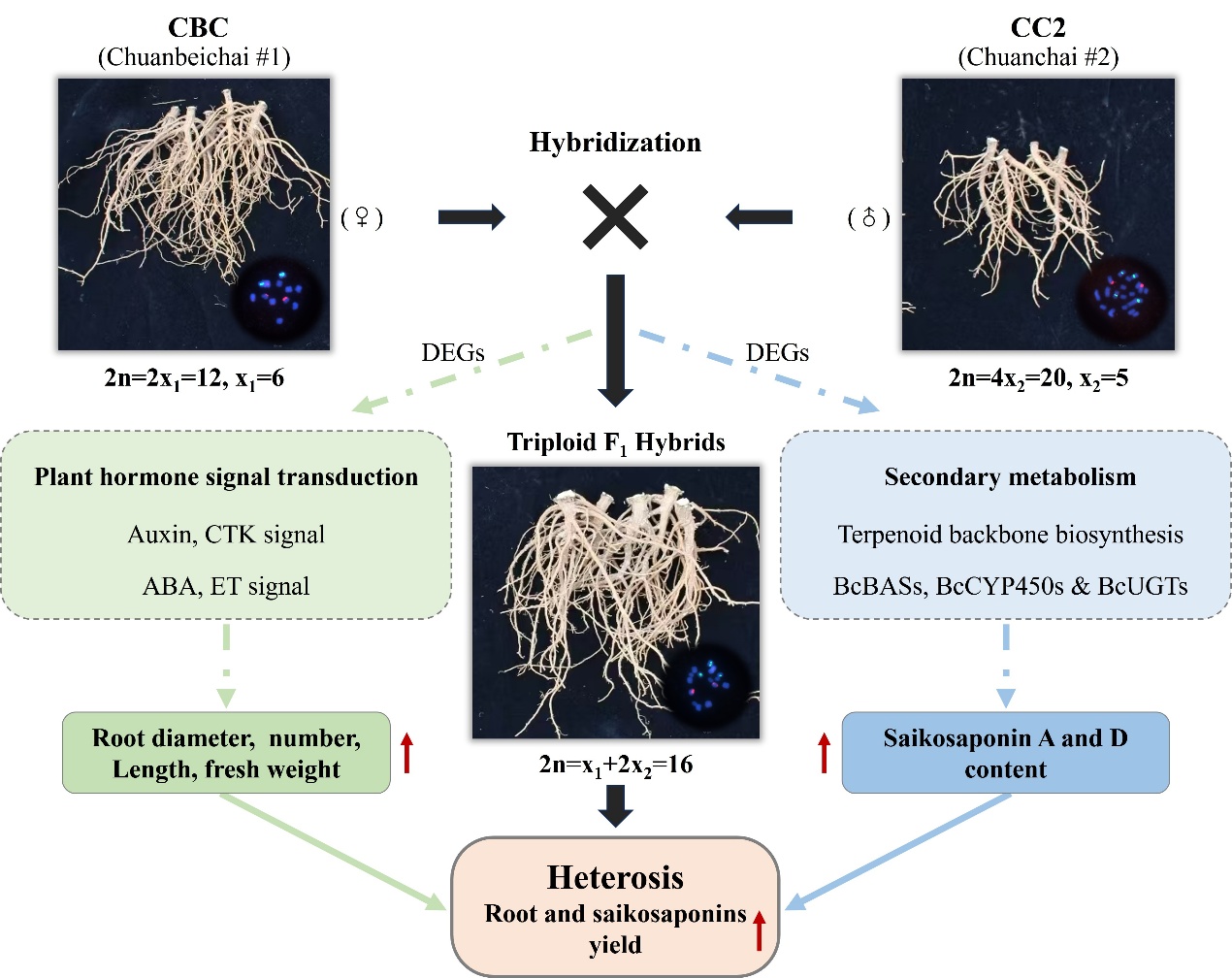


**FIGURE S8.** Mechanism diagram of the triploid F1 generation of *B. chinense.*
