# Supplementary material for: Heterosis unveiled in root-related traits and saikosaponins content between triploid F1 hybrids and parental Bupleurum chinense DC
Source: Front Plant Sci. 2026 Feb 12;17:1736464. doi: 10.3389/fpls.2026.1736464 (PMC12935959; doi:10.3389/fpls.2026.1736464)
Supplement: Supplementary file 2 [file Table1.docx]

**TABLE S1.** Primer sequence for the selected genes in qRT-PCR

| **Gene ID** | **Gene name** | **Forward primer (5’ → 3’)** | **Reverse primer sequence(5’ → 3’)** |
| --- | --- | --- | --- |
| *Hap1_chr1G8281* | *BcIAA13.1* | CTGGGACTTGGCCTGAGC | GGCAACCTCAGGAGAAGCA |
| *Hap1_chr4G363* | *BcSAUR24.1* | AAGGCTGTGTGGCGGTTT | TATGGTCAAGCCTCCGCG |
| *Hap1_chr3G7452* | *BcCYP716A83.1* | TGGATGGCCCATCATCGG | AAAACTGCTGCCGGCTCT |
| *Hap1_chr5G3928* | *BcCYP716Y1.1* | GCCCCGCGTTTTACCCAT | AGGCGCACCATCACCTTC |
| *Hap1_chr1G9522* | *BcUGT73.1* | CTCCTGGCCACCTCATGC | AAGGCCCGAATCGACAGC |
| *Hap1_chr3G5569* | *BcUGT74.1* | TACCTGGGGCTGCCTTCT | TTTGGTGGCGGCAATCCT |

**TABLE S2.** The mean value, standard error and significant comparisons (sig) results among CBC, F1 and CC2.

| Trait | Genotype | Seedling | | |  | Maturity (2021) | | |  | Maturity (2022) | | |
| --- | --- | --- | --- | --- | --- | --- | --- | --- | --- | --- | --- | --- |
|  |  | Mean | SE | sig |  | Mean | SE | sig |  | Mean | SE | sig |
| Plant height (cm) | CBC | - | - | - |  | 90.3 | 1.42 | c |  | 82.49 | 0.6 | c |
|  | F1 | - | - | - |  | 130.3 | 0.97 | b |  | 114.79 | 3.2 | b |
|  | CC2 | - | - | - |  | 161.4 | 0.70 | a |  | 142.91 | 1.71 | a |
| Root length (cm) | CBC | 13.05 | 0.48 | a |  | 12.8 | 0.55 | ab |  | 16.50 | 0.76 | a |
|  | F1 | 12.72 | 0.40 | a |  | 13.4 | 0.44 | a |  | 15.54 | 0.66 | b |
|  | CC2 | 13.82 | 1.23 | a |  | 11.8 | 0.12 | b |  | 14.16 | 0.42 | b |
| Lateral root number | CBC | 25 | 0.83 | b |  | 8 | 0.56 | c |  | 10 | 0.06 | a |
|  | F1 | 29 | 1.17 | a |  | 12 | 0.27 | a |  | 12 | 0.61 | a |
|  | CC2 | 10 | 0.70 | c |  | 10 | 0.62 | b |  | 11 | 1.07 | a |
| Root diameter (mm) | CBC | 2.57 | 0.06 | b |  | 7.68 | 0.28 | b |  | 6.84 | 0.18 | b |
|  | F1 | 3.23 | 0.07 | a |  | 10.64 | 0.13 | a |  | 7.95 | 0.10 | a |
|  | CC2 | 1.78 | 0.04 | c |  | 6.79 | 0.05 | c |  | 3.40 | 0.35 | c |
| Fresh root weight (g) | CBC | 0.67 | 0.14 | b |  | 4.80 | 0.07 | b |  | 4.64 | 0.06 | b |
|  | F1 | 1.32 | 0.26 | a |  | 6.95 | 0.46 | a |  | 6.03 | 0.07 | a |
|  | CC2 | 0.29 | 0.08 | b |  | 2.37 | 0.13 | c |  | 2.70 | 0.03 | c |
| Dry root weight (g) | CBC | 0.09 | 0.02 | b |  | 1.81 | 0.03 | b |  | 1.84 | 0.04 | b |
|  | F1 | 0.17 | 0.03 | a |  | 2.43 | 0.16 | a |  | 2.58 | 0.07 | a |
|  | CC2 | 0.04 | 0.01 | b |  | 0.90 | 0.05 | c |  | 0.98 | 0.03 | c |
| Saikosaponin A (mg/g) | CBC | 3.95 | 0.40 | a |  | 3.34 | 0.04 | c |  | 5.78 | 0.14 | c |
|  | F1 | 3.78 | 0.43 | a |  | 3.86 | 0.19 | b |  | 6.75 | 0.19 | b |
|  | CC2 | 2.47 | 0.31 | b |  | 5.05 | 0.09 | a |  | 8.29 | 0.02 | a |
| Saikosaponin D (mg/g) | CBC | 2.37 | 0.29 | b |  | 3.43 | 0.10 | c |  | 5.71 | 0.08 | c |
|  | F1 | 2.55 | 0.33 | a |  | 4.01 | 0.15 | b |  | 6.44 | 0.16 | b |
|  | CC2 | 1.28 | 0.28 | c |  | 4.89 | 0.11 | a |  | 8.71 | 0.07 | a |
| Total saikosaponin A (mg/strain) | CBC | 0.34 | 0.07 | b |  | 6.04 | 0.12 | b |  | 10.66 | 0.47 | b |
|  | F1 | 0.61 | 0.12 | a |  | 9.38 | 0.67 | a |  | 17.46 | 0.84 | a |
|  | CC2 | 0.10 | 0.03 | c |  | 4.56 | 0.24 | c |  | 8.15 | 0.25 | c |
| Total saikosaponin D (mg/strain) | CBC | 0.20 | 0.04 | b |  | 6.22 | 0.21 | b |  | 10.53 | 0.34 | b |
|  | F1 | 0.40 | 0.08 | a |  | 9.73 | 0.48 | a |  | 16.66 | 0.77 | a |
|  | CC2 | 0.05 | 0.01 | c |  | 4.42 | 0.26 | c |  | 8.56 | 0.32 | c |

**TABLE S3.** Sample name, total pairs, sample size, Q30 value and mapped ratio of the transcriptome among the maturity root samples of CBC, F1 and CC2.

| **Stage** | **Samples** | **Samples ID** | **Size (GB)** | **Total pairs** | **GC (%)** | **Q30 (%)** | **mapped ratio (%)** |  |
| --- | --- | --- | --- | --- | --- | --- | --- | --- |
|  |  |  |  |  |  |  |  |  |
| Seedling | CBC_1 | CBC_1.R1.fq.gz | 1.46 | 26616671 | 43.28 | 95.96 | 82.16 |  |
|  |  | CBC_1.R2.fq.gz | 1.51 |  | 43.35 | 95.58 |  |  |
|  | CBC_2 | CBC_2.R1.fq.gz | 1.44 | 29220596 | 43.18 | 96.14 | 81.90 |  |
|  |  | CBC_2.R2.fq.gz | 1.48 |  | 43.28 | 95.64 |  |  |
|  | CBC_3 | CBC_3.R1.fq.gz | 1.49 | 30287889 | 43.29 | 96.06 | 81.81 |  |
|  |  | CBC_3.R2.fq.gz | 1.52 |  | 43.35 | 95.73 |  |  |
|  | F1_1 | F1_1.R1.fq.gz | 1.76 | 26961066 | 43.11 | 96.21 | 85.25 |  |
|  |  | F1_1.R2.fq.gz | 1.81 |  | 43.13 | 96.57 |  |  |
|  | F1_2 | F1_2.R1.fq.gz | 1.42 | 25659029 | 43.04 | 95.94 | 85.17 |  |
|  |  | F1_2.R2.fq.gz | 1.43 |  | 43.06 | 96.35 |  |  |
|  | F1_3 | F1_3.R1.fq.gz | 1.43 | 28549640 | 43.04 | 95.91 | 85.06 |  |
|  |  | F1_3.R2.fq.gz | 1.48 |  | 43.10 | 95.51 |  |  |
|  | CC2_1 | CC2_1.R1.fq.gz | 1.40 | 28175746 | 42.93 | 96.25 | 89.04 |  |
|  |  | CC2_1.R2.fq.gz | 1.44 |  | 42.95 | 96.61 |  |  |
|  | CC2_2 | CC2_2.R1.fq.gz | 1.59 | 23668901 | 42.86 | 96.21 | 89.28 |  |
|  |  | CC2_2.R2.fq.gz | 1.64 |  | 42.89 | 96.32 |  |  |
|  | CC2_3 | CC2_3.R1.fq.gz | 1.37 | 23503539 | 43.03 | 96.12 | 89.54 |  |
|  |  | CC2_3.R2.fq.gz | 1.42 |  | 43.05 | 96.46 |  |  |
| Maturity | CBC_1.x | CBC_1.x.R1.fq.gz | 1.46 | 22073061 | 42.42 | 95.63 | 80.39 |  |
|  |  | CBC_1.x.R2.fq.gz | 1.51 |  | 42.43 | 94.33 |  |  |
|  | CBC_2.x | CBC_2.x.R1.fq.gz | 1.44 | 21693424 | 42.38 | 95.45 | 80.00 |  |
|  |  | CBC_2.x.R2.fq.gz | 1.48 |  | 42.39 | 94.50 |  |  |
|  | CBC_3.x | CBC_3.x.R1.fq.gz | 1.49 | 22540244 | 42.46 | 95.63 | 80.57 |  |
|  |  | CBC_3.x.R2.fq.gz | 1.52 |  | 42.46 | 94.92 |  |  |
|  | F1_1.x | F1_1.x.R1.fq.gz | 1.76 | 26734558 | 42.41 | 95.64 | 85.48 |  |
|  |  | F1_1.x.R2.fq.gz | 1.81 |  | 42.41 | 94.69 |  |  |
|  | F1_2.x | F1_2.x.R1.fq.gz | 1.42 | 21353166 | 42.44 | 95.44 | 85.57 |  |
|  |  | F1_2.x.R2.fq.gz | 1.43 |  | 42.45 | 95.26 |  |  |
|  | F1_3.x | F1_3.x.R1.fq.gz | 1.43 | 21711156 | 42.51 | 95.62 | 84.79 |  |
|  |  | F1_3.x.R2.fq.gz | 1.48 |  | 42.51 | 94.59 |  |  |
|  | CC2_1.x | CC2_1.x.R1.fq.gz | 1.40 | 21153461 | 42.30 | 95.39 | 88.99 |  |
|  |  | CC2_1.x.R2.fq.gz | 1.44 |  | 42.31 | 94.61 |  |  |
|  | CC2_2.x | CC2_2.x.R1.fq.gz | 1.59 | 23784690 | 42.68 | 95.90 | 89.55 |  |
|  |  | CC2_2.x.R2.fq.gz | 1.64 |  | 42.69 | 94.81 |  |  |
|  | CC2_3.x | CC2_3.x.R1.fq.gz | 1.37 | 20647610 | 42.21 | 95.43 | 91.16 |  |
|  |  | CC2_3.x.R2.fq.gz | 1.42 |  | 42.22 | 94.29 |  |  |
